# Controlled Ligand Exchange Between Ruthenium Organometallic Cofactor Precursors and a Naïve Protein Scaffold Generates Artificial Metalloenzymes Catalysing Transfer Hydrogenation

**DOI:** 10.1002/anie.202015834

**Published:** 2021-03-26

**Authors:** George S. Biggs, Oskar James Klein, Sarah L. Maslen, J. Mark Skehel, Trevor J. Rutherford, Stefan M. V. Freund, Florian Hollfelder, Sally R. Boss, Paul D. Barker

**Affiliations:** ^1^ Department of Chemistry University of Cambridge Lensfield Road Cambridge CB2 1EW UK; ^2^ Department of Biochemistry University of Cambridge Tennis Court Road Cambridge CB2 1GA UK; ^3^ MRC Laboratory of Molecular Biology Francis Crick Avenue, Cambridge Biomedical Campus Cambridge CB2 0QH UK

**Keywords:** direct coordination, ligand exchange, metalloenzymes, ruthenium, transfer hydrogenation

## Abstract

Many natural metalloenzymes assemble from proteins and biosynthesised complexes, generating potent catalysts by changing metal coordination. Here we adopt the same strategy to generate artificial metalloenzymes (ArMs) using ligand exchange to unmask catalytic activity. By systematically testing Ru^II^(η^6^‐arene)(bipyridine) complexes designed to facilitate the displacement of functionalised bipyridines, we develop a fast and robust procedure for generating new enzymes via ligand exchange in a protein that has not evolved to bind such a complex. The resulting metal cofactors form peptidic coordination bonds but also retain a non‐biological ligand. Tandem mass spectrometry and ^19^F NMR spectroscopy were used to characterise the organometallic cofactors and identify the protein‐derived ligands. By introduction of ruthenium cofactors into a 4‐helical bundle, transfer hydrogenation catalysts were generated that displayed a 35‐fold rate increase when compared to the respective small molecule reaction in solution.

## Graphical abstract

A ruthenium organometallic complex is transformed into an effective transfer hydrogenation catalyst upon exchanging ligands with a naïve protein. The direct coordination of protein sidechains to the metal is an underutilised feature in artificial metalloenzymes.

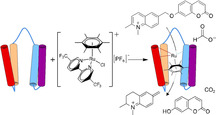

## Introduction

Creating artificial metalloenzymes from transition metal cofactors embedded in proteins allows for expansion of nature's catalytic repertoire, bringing about new‐to‐nature reactivity.[^1^, ^2^, ^3^, ^4^, ^5^, ^6^, ^7^, ^8^, ^9^, ^10^] Catalysts of remarkable activity and selectivity have been obtained and evolved with naturally‐biosynthesised metal cofactors and bare metal ions by varying the protein component of ArMs.[^11^, ^12^, ^13^, ^14^, ^15^, ^16^, ^17^, ^18^] However, comparable levels of evolutionary rate enhancement have yet to be achieved when using organometallic cofactors.[^19^, ^20^, ^21^, ^22^, ^23^, ^24^, ^25^, ^26^, ^27^, ^28^] By expanding the reaction scope of enzymes to include catalytic activities normally exclusive to small molecules, ArMs have the potential to achieve efficient transformations without the need for activated substrates bearing directing groups, as commonly encountered in classic asymmetric small molecule catalysis. Additionally, ArMs are fully functional in aqueous solution, making it possible to avoid toxic organic solvents, which is a key advance towards more sustainable, green chemistry.[^29^, ^30^] Already, “biofoundries” can be envisioned for synthesising or modifying essentially any conceivable organic molecule by pathway engineering using natural enzymes.^[31]^ The addition of orthogonal transition metal activity to the synthetic biologists’ toolbox would open up alternative metabolic routes with fewer steps, and better carbon and energy efficiency.^[32]^

Strategies for generating ArMs containing non‐natural metal cofactors include: metal substitution,[^25^, ^26^, ^33^, ^34^, ^35^, ^36^] supramolecular assembly,[^5^, ^24^, ^37^, ^38^, ^39^] covalent attachment,[^22^, ^40^, ^41^, ^42^, ^43^, ^44^] and coordinative metal‐protein bonding.[^13^, ^15^, ^45^, ^46^, ^47^, ^48^, ^49^, ^50^] However, an additional distinction can be made based on whether the ArM′s metal cofactor contains a metal‐protein coordination bond in the holoprotein or not. In the majority of ArMs published to date, such a coordination bond is not formed, which crucially affects function and evolvability.[^19^, ^27^, ^51^] Without a metal‐protein coordination bond, the first coordination sphere of the protein‐bound metal cofactor (i.e. the coordinated ligands) is unchanged from the precursor complex in solution. While this does not preclude a suitable protein scaffold from being evolved by improved binding arrangements vs. the target substrates, the core catalytic moiety will only be affected indirectly as any changes to the protein are restricted to the second coordination sphere.

Systems containing metal‐peptide bonds, or dative ArMs, have been described, but mainly contain bare metal ions.[^13^, ^15^, ^50^] This has both biological and chemical consequences. Firstly, cells tend to enact stringent control over bare metal ions in solution, making a conjugation system reliant on uptake of solvated ions difficult to translate into a living host.^[52]^ Secondly, the limited ligand set available to natural proteins may restrict the type of catalysis that can be achieved, although this can be circumvented by the use of unnatural amino acids. Both issues could be alleviated by using an organometallic complex as a precursor to the reactive, protein‐bound cofactor.

**Figure 1:**
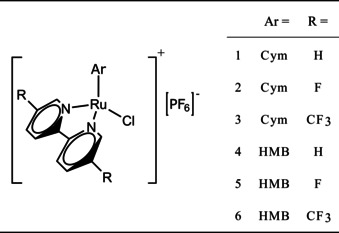
Simplified structures of complexes of the form [Ru^II^(η^6^‐arene)(bipyridine)Cl]^+^[PF_6_]^−^. Ar denotes different arene substituents, R denotes different substituents on the 5,5′‐position on the bipyridine. Bipyridine=Bipy, 5,5′‐difluorobipyridine=FBipy, 5,5′‐trifluoromethylbipyridine=TFMBipy, arene=p‐cymene (Cym) or hexamethylbenzene (HMB).

Here we set out to generate protein‐metal conjugates by attaching small‐molecule ruthenium complexes to protein scaffolds through ligand exchange, forming a cofactor which is embedded within a structured environment. By systematically enhancing the ability of chelating ligands on the metal to be displaced upon binding to the protein, organometallic cofactors containing multiple protein‐metal coordination bonds were successfully formed in a controlled fashion. The resulting metal‐protein conjugates that combine cytochrome *b*_562_ variants with a suite of Ru^II^(η^6^‐arene)(bipyridine) complexes (Figure 1) show activity as catalysts for transfer hydrogenation.

## Results and Discussion

### Controlled Protein Conjugation via Ligand Exchange

**Figure 2:**
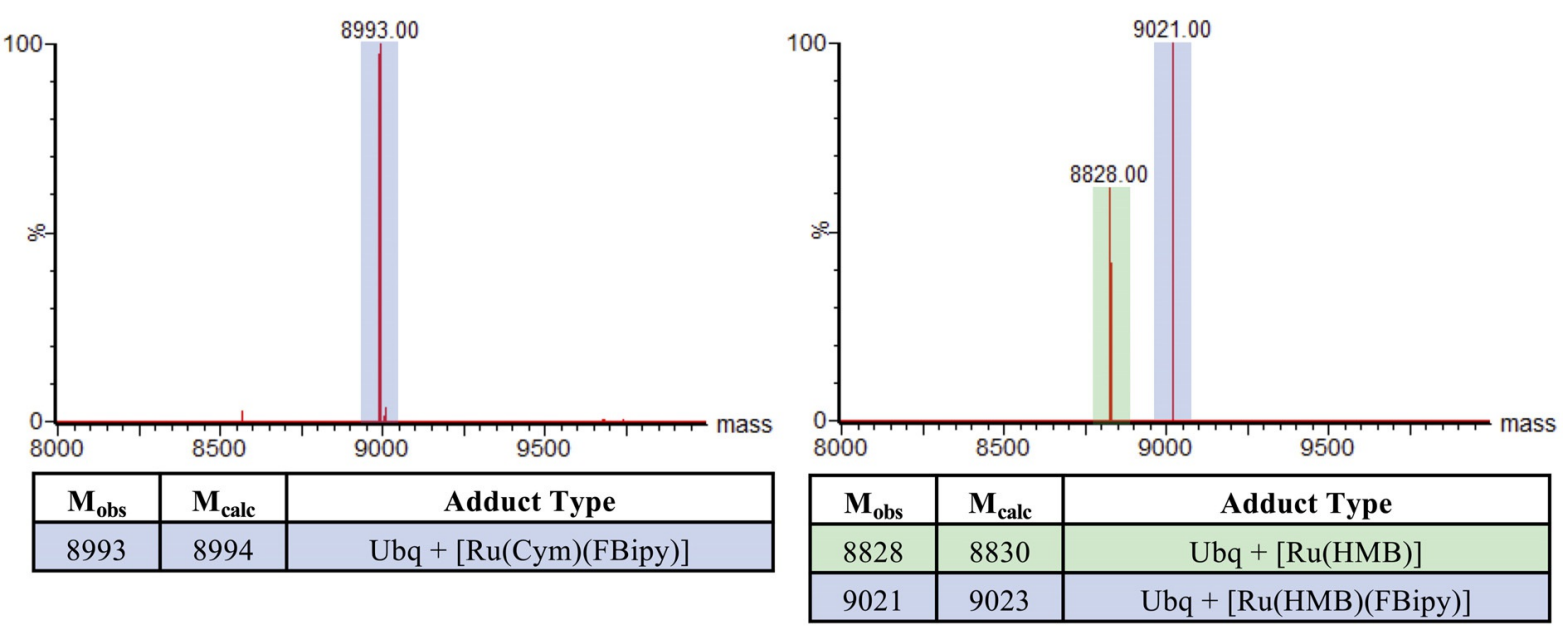
Mass spectra from incubations of complex [Ru^II^(Cym)(FBipy)Cl]^+^ (Left) and complex [Ru^II^(HMB)(FBipy)Cl]^+^ (Right) with Ubq K63C (50 μM protein, 20 Eq. Ru complex, 37 °C, 1 Hr).

We have previously used ^19^F NMR spectroscopy to directly report the behaviour in aqueous solution of organometallic complexes carrying fluorinated ligands.^[53]^ Specifically, the ligand exchange between a suite of [Ru^II^(arene)(bipyridine)Cl]^+^ complexes and mixtures of amino acids established the cysteine thiol as the thermodynamically‐preferred replacement for the labile monodentate chloride ligand. Expanding upon the work with small molecules we explored the binding of these complexes to proteins and monitored their speciation through a combination of LC‐MS and ^19^F NMR spectroscopy. Ubiquitin K63C and cytochrome b_562_ L10C/H102M have accessible cysteine residues for anchoring ruthenium complexes via ligand exchange and thus were examined initially. Indeed, when the single cysteine‐containing ubiquitin mutant, Ubq K63C, was incubated with [Ru^II^(η^6^‐cymene)(5,5′‐difluorobipyridine)Cl]^+^, [2], a single species was observed by LC‐MS, where the protein was modified with the metal fragment [Ru(Cym)(FBipy)] (highlighted blue in Figure 2).

In contrast, after incubation with complex [5], an additional species was observed corresponding to Ubq K63C + [Ru(HMB)] (highlighted green in Figure 2), indicating loss of the bipyridyl ligand. Subsequent incubation of Ru‐Ubq hybrids with N‐ethyl maleimide (NEM) did not lead to any further changes in mass, confirming that the thiol was not free to react and verifying that these metal complexes prefer to bind to thiols, Figure S1. As the mass of the Ubq K63C + [Ru(HMB)] adduct did not indicate the coordination of further small molecules for example, solvent, or ions to the metal, these observations suggest that the newly‐assembled holoprotein complex had been formed by cysteine coordination, but with additional peptidic ligands replacing bipyridine in the first coordination sphere of the metal ion. The bipyridine‐metal coordination linkage is stable in water and amino acid solutions, but dissociation from the metal can clearly be facilitated in a protein context favouring additional exchange. Importantly, the newly generated complex had to be formed by relatively weakly coordinating peptide‐derived ligands (protein backbone, or sidechain carboxylates, amides, alcohols, phenols, amines, imidazoles). This may be key for catalysis as, with more labile ligands, the metal centre can undergo subsequent ligand exchange with a substrate to initiate a catalytic cycle (vide infra).

**Figure 3:**
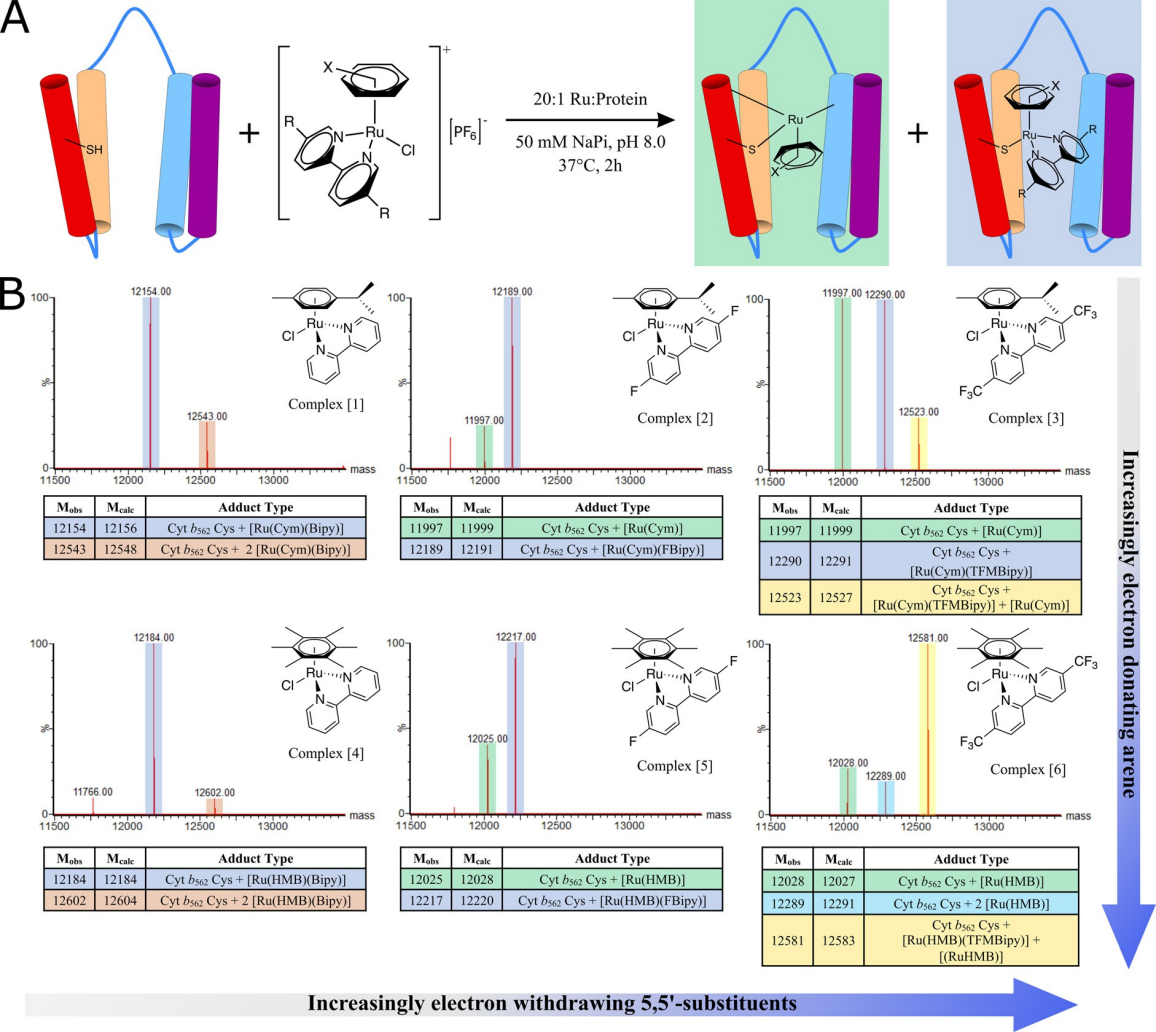
**(A)** Simplified reaction scheme of Cyt *b*_562_ Cys with the Ru(arene)(bipyridine) complexes [1]–[6] to show the singly modified protein‐metal hybrids generated in the incubations. **(B)** Mass spectra from incubations of Cyt *b*_562_ Cys with complexes [1]–[6] (50 μM protein, 20 Eq. Ru, 37 °C, 1 Hr, *x*‐axis: mass between 11 500 and 14 000 mass units, *y*‐axis: signal intensity as % of max). The structures of the singly charged complexes (PF_6_ removed) added at the start of the incubation are overlaid, and the assignments of adducts are given in the tables below the spectra.

The observed loss of bipyridine ligand upon protein conjugation demonstrated the potential for [5] and similar complexes to undergo extensive ligand exchange with the protein beyond the single, monodentate exchange observed using amino acids in solution. Further use of ubiquitin was thought to be unlikely to provide a promising scaffold for a putative ArM given its small size and lack of a well‐defined or nascent hydrophobic pocket leading to a high probability of the metal cofactor being bound to the surface. This would inhibit an ArM to selectively bind and organise substrates. Furthermore, as it was not possible to isolate the Ubq K63C + [Ru(HMB)] conjugate from the bipyridine‐coordinated analogue for characterisation and further catalytic studies, a more detailed study was undertaken using a suite of related complexes [1]–[6] to explore the speciation within the more promising context of cytochrome *b*_562_. The speciation patterns on incubation of these complexes with the four‐helix bundle protein cytochrome *b*_562_, L10C/H102M are shown in Figure 3. The choice of organometallic cofactor with general formula [Ru^II^(η^6^‐arene)(bipyridine)Cl]^+^ can be rationalised in terms of a balance of aqueous solubility and stability as well as latent ligand lability. The monodentate chloride ligand is readily replaceable by water and, depending on pH, generates a Ru‐OH_2_/OH bond which is in turn labilised on formation of a primary coordination link to the protein.[^54^, ^55^]

The arene component of the complex confers stability on the Ru^II^ oxidation state and can be easily tuned to modulate electron density and steric pressure on the metal.^[56]^ The HMB ligand is more electron donating and a greater steric presence than Cym. Incorporation of fluorine atoms into the bipyridine ligand also enables electronic tuning with the electron withdrawing ability increasing from Bipy to FBipy to TFMBipy. The loss of either arene or bipyridine is expected to be slow for these complexes, given their chelating nature and ruthenium's slow metal‐ligand exchange rates.^[57]^ Complexes can therefore be predicted to be stable to ligand exchange in aqueous solution but become more labile upon equilibration with the protein, via the formation of coordination links and/or non‐covalent interactions within the protein scaffold. These organometallic complexes were designed to promote displacement of the bipyridine by the protein in a controlled manner, therefore finding a balance between ArM yield and non‐specifically modified protein as an effect of the sequentially more electron withdrawing 5,5′‐substituents on the bipyridine and the steric and electronic contributions of the arene. Cytochrome *b*_562_ was chosen for its folding dynamics as well as its nascent haem cofactor binding site. In the apoprotein form, this four‐helical‐bundle protein is in dynamic, partially folded states, with complete folding only being initiated by association of the heme cofactor.[^58^, ^59^, ^60^] This makes the four‐helix‐bundle protein highly versatile and promiscuous towards accommodating different cofactors and a logical starting point for inclusion of an organometallic cofactor.^[61]^ The mutant four‐helix bundle protein cytochrome *b*_562_, L10C/H102M (henceforth referred to as “Cyt *b*_562_ Cys”) had been historically designed for the covalent attachment of heme at the cysteine residue.^[62]^

The reactions of complexes [1]–[6] with Cyt *b*_562_ Cys showed that tuning the reactivity of the organometallic complexes had an effect on speciation. Whereas no bipyridine dissociation was observed using 5,5′‐H‐bipyridine (complexes [1] and [4], highlighted blue in Figure 3), increasing lability was observed across the series, culminating in much increased bipyridine dissociation when the substituents were ‐CF_3_, that is, complexes [3] and [6]. In addition to the mono‐metallated conjugates, di‐metallated species were also observed for [1], [3], [4] and [6]. Single modification of the protein was favoured by reducing the metal complex to protein ratio and incubation time, two further means of influencing metal‐protein speciation.

Those metal‐protein conjugates which contained a cofactor carrying a bipyridine ligand were isolated via anion exchange chromatography. Products obtained from incubations of Cyt *b*_562_ Cys with [2], [3], [5] & [6] did not react with N‐ethyl maleimide and all contained fluorinated ligands, enabling structural assignment via ^19^F NMR spectroscopy. The chemical shift values for the fluorine atoms were consistent with cysteine coordination (Figure S2).^[53]^ Additionally, the spectra displayed two distinct ^19^F resonances with different linewidths, with each peak corresponding to one of the two fluorine atoms on the bipyridine ligand. The different shifts and relaxation properties attributed to the individual atoms suggested the fluorine atoms were situated in distinct chemical environments within the protein, potentially distinguished by buried or solvent‐exposed positionings, thus highlighting the ability of the scaffold to create asymmetric conditions for an unnatural cofactor, a principal demand for stereoselective catalysis.

After establishing speciation, the catalytic potential of these hybrids was explored via a transfer hydrogenation assay. This reaction is known to be Ru^II^ catalysed, has a well understood mechanism and was chosen due to its wide range of potential applications.[^63^, ^64^, ^65^] The mechanism of transfer hydrogenation does not necessitate a change in metal oxidation state hence the rate is dependent on ligand exchange kinetics, rather than redox chemistry. For catalysis to occur at the metal centre, a ligand must exchange with a hydrogen donor (e.g. formate) to form a ruthenium‐hydride species, which can then hydrogenate the substrate. Hydrogenation was monitored via the reduction of a pre‐fluorescent quinolinium substrate **1** developed by Ward et al. (Figure S3 & S4).^[66]^

For those hybrids where bipyridine dissociation had not occurred (highlighted blue in Figure 3), there was no observable catalytic activity. In these cases, the metal cofactor appeared to maintain a stable first coordination sphere, suggesting that its stability precludes activity. Where protein metallation led to cysteine coordination with loss of bipyridine (highlighted green in Figure 3), the potential for the protein to provide multiple peptidic ligands opened up the possibility for catalysis. Unfortunately, isolating the desired species, the 1:1 adducts of Cyt *b*_562_ Cys and [Ru(arene)], from the incubation mixtures was not readily achieved.

The reactions with the cysteine variant had demonstrated that changing the electronic properties of the ligands influenced the speciation significantly. For the metallation reactions presented, all approached completion rapidly and could also be achieved with only minor excess of metal complex. However, the reaction yielded a mixture of protein‐metal hybrids, with and without bipyridine dissociation which could not be individually isolated. Based on these preliminary findings it was hypothesised, that different, more dynamic speciation should be explored using a protein scaffold without the very rapidly reacting cysteine, as this could potentially be trapping the newly formed metalloprotein in a conformation where the bipyridine cannot be displaced.[^37^, ^39^, ^67^] Instead, a scaffold with reduced positional dependency on a single cysteine residue was chosen, allowing for equilibration of the cofactor and protein during the reaction. Therefore, the same set of complexes [1]–[6] were incubated with the cysteine‐free, wild‐type cytochrome *b*_562_ (Cyt *b*_562_ wt).

**Figure 4:**
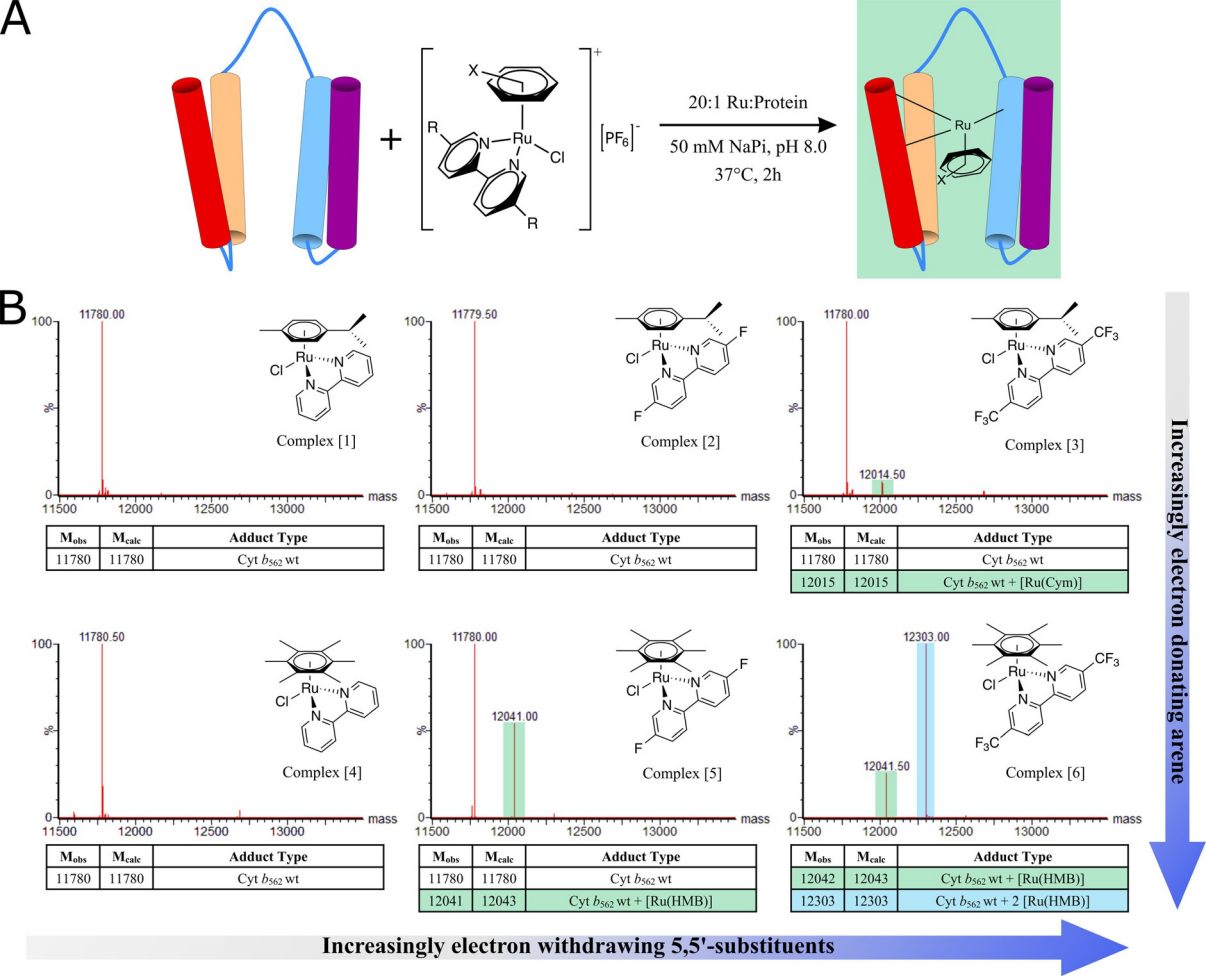
**(A)** Simplified reaction scheme of Cyt *b*_562_ wt with the Ru(arene)(bipyridine) complexes [1]–[6] to show the singly modified protein‐metal hybrids generated in the incubations. **(B)** Mass spectra from incubations of Cyt *b*_562_ wt with complexes [1]–[6] (50 μM protein, 20 Eq. Ru, 37 °C, 1 Hr, *x*‐axis: mass between 11 500 and 14 000 mass units, *y*‐axis: signal intensity as % of max). The structures of the singly charged complexes (PF_6_ removed) added at the start of the incubation are overlaid, and the assignments of adducts are given in the tables below the spectra.

As work with amino acids had shown,^[53]^ these same ruthenium complexes do undergo ligand exchange with Lewis basic amino acid functionalities other than thiol—albeit with a much lower affinity—and therefore different speciation was expected from these incubations. Indeed, complexes [1, 2 & 4] did not produce any observable modified protein in the absence of the strong thiol ligand (Figure 4). However, the more active complexes [3 & 5] produced a single mono‐substituted hybrid protein (identified by mass spectrometry, highlighted green in Figure 4), where loss of the bipyridine had occurred. Incubation with [6] resulted in complete conversion with considerable quantities of a doubly‐modified protein also observed (highlighted cyan in Figure 4). By reducing the number of equivalents of ruthenium complexes added from 20 to 2, full modification of Cyt *b*_562_ wt was achieved within 2 hours with a strong preference for the desired mono‐substituted variant.

Having established a simple, yet highly efficient, method of generating hybrid proteins via ligand exchange, efforts were made to isolate and characterise these novel metalloproteins. Anion‐exchange chromatography proved to be an effective method, however, it was observed that, instead of purifying into a single fraction, the mono‐substituted Cyt *b*_562_ wt + [Ru(HMB)] adducts separated into two fractions (Figure S5). These fractions contained a protein of the same mass but upon closer inspection showed clearly distinct ion series in their ESI‐MS spectra. These individual fractions did not appear to interconvert in buffer after weeks nor after buffer exchange, ruling out dynamic causes such as different protonation states or interchangeable conformation. Thus, the fractions were henceforth treated as separate variants, denoted as Hybrid 1 & 2 and Hybrids 3 & 4 for the products of incubation of the protein with [5] and [6] respectively, Table 1.

**Table 1:** Origin and analysis of the protein metal hybrids isolated from incubations of Cyt *b*_562_ wt and complexes [5] and [6]. Retention time is given for anion exchange, see Figure S5.

Hybrid	Incubation	*M*_obs_	Retention Time (mins)	*T*_m_ (°C)	Highest Intensity MS Peak
1	Cyt *b*_562_ wt + 20 Eq. [5]	12 042	7.6	63.4	+11 (1095.65)
					
2	Cyt *b*_562_ wt + 20 Eq. [5]	12 042	9.8	59.4	+14 (861.15)
					
3	Cyt *b*_562_ wt + 2 Eq. [6]	12 042	7.6	64.0	+11 (1095.65)
					
4	Cyt *b*_562_ wt + 2 Eq. [6]	12 042	9.8	57.8	+14 (861.15)

### Catalytic Activity of Ruthenium–Cyt b_562_ wt Hybrids

**Figure 5:**
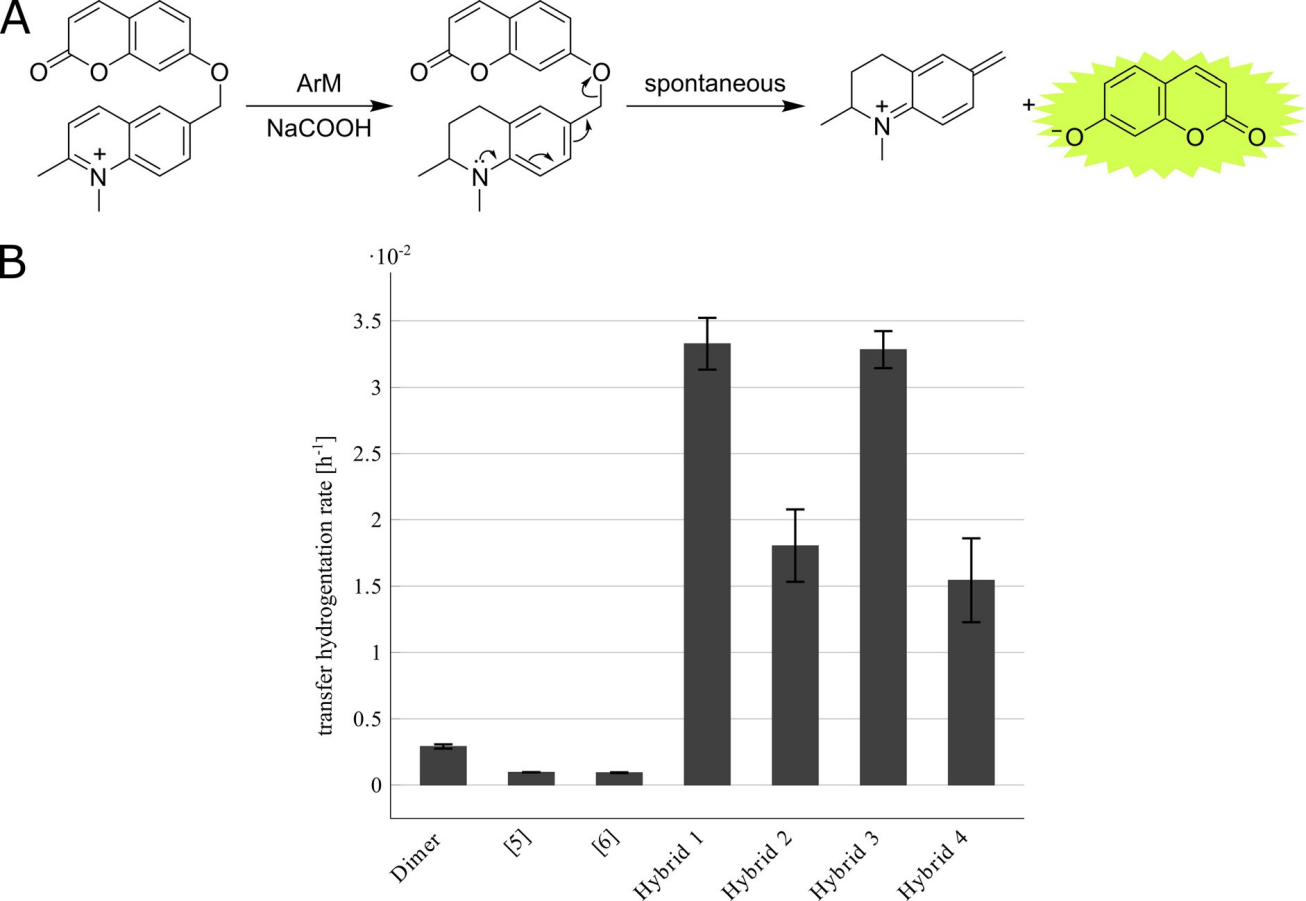
**(A)** Scheme for the transfer hydrogenation reaction used to monitor catalytic activity. Upon reduction of the quinoline moiety the molecule undergoes spontaneous self‐immolation to release the fluorescent umbelliferone. **(B)** Rate of transfer hydrogenation for the Cyt *b*_562_–Ru Hybrids 1–4, the Ru‐HMB complexes [5] and [6], and the known dimeric catalyst [Ru(Cym)Cl_2_]_2_. The rates have been measured and normalised by ruthenium concentration. Error bars are one standard error of the mean calculated from at least 3 independent repeats. *Conditions*: 50 mM sodium phosphate, pH 8.0, 100 mM formate, 1 mM substrate **1**, measured over 16 h at 37 °C. Typical individual time traces are given in Figure S3 & S4.

Satisfyingly, all hybrid variants, Hybrids 1–4, displayed significant activity in the transfer hydrogenation assay, establishing these proteins as ArMs, Figure 5. Noticeably, Hybrids 1 and 3 had the same activity, even though they were derived from different complexes, as did Hybrids 2 and 4 (see Figure S5), with the earlier eluting species, Hybrids 1 and 3, being slightly more active. Compared to the well‐established, small molecule catalyst, dichloro(*p*‐cymene)ruthenium(II) dimer, [Ru(Cym)Cl_2_]_2_, the rates of transfer hydrogenation were ten‐fold greater for Hybrids 1 and 3. Importantly, ligand exchange with the protein yielded an up to 35‐fold increase in rate compared to the small‐molecule ruthenium complexes, [5] and [6] respectively.

In order to gain a more direct measure of the rate of metallation, the same assay was employed to monitor the metal‐protein conjugation reaction in situ by measuring the transfer hydrogenation activity over time after addition of one equivalent of complex [6] to Cyt *b*_562_ wt. The kinetic traces obtained initially followed the same rate as the free metal complex before gradually increasing in activity as the ArM is formed in situ until full conversion into hybrid protein was reached and a constant rate is observed, as indicated by a linear trace (Figure S6). Using equimolar amounts, complete conjugation of Cyt *b*_562_ wt was achieved in less than two hours at 37 °C. Mass spectrometry analysis confirmed that the mono‐substituted hybrid variants were the dominant species with minor contamination by some di‐substituted protein. Further, the maximum rate observed was in accordance with those obtained from the purified samples. Thus, similar activities to that from isolated species can be achieved in a single reaction vessel without purification, demonstrating the viability of “one‐pot” ArMs generated by ligand exchange reactions, to be used in techniques where individual purification may be limiting.

### Characterisation of Ruthenium–Cyt b_562_ wt Hybrids

**Figure 6:**
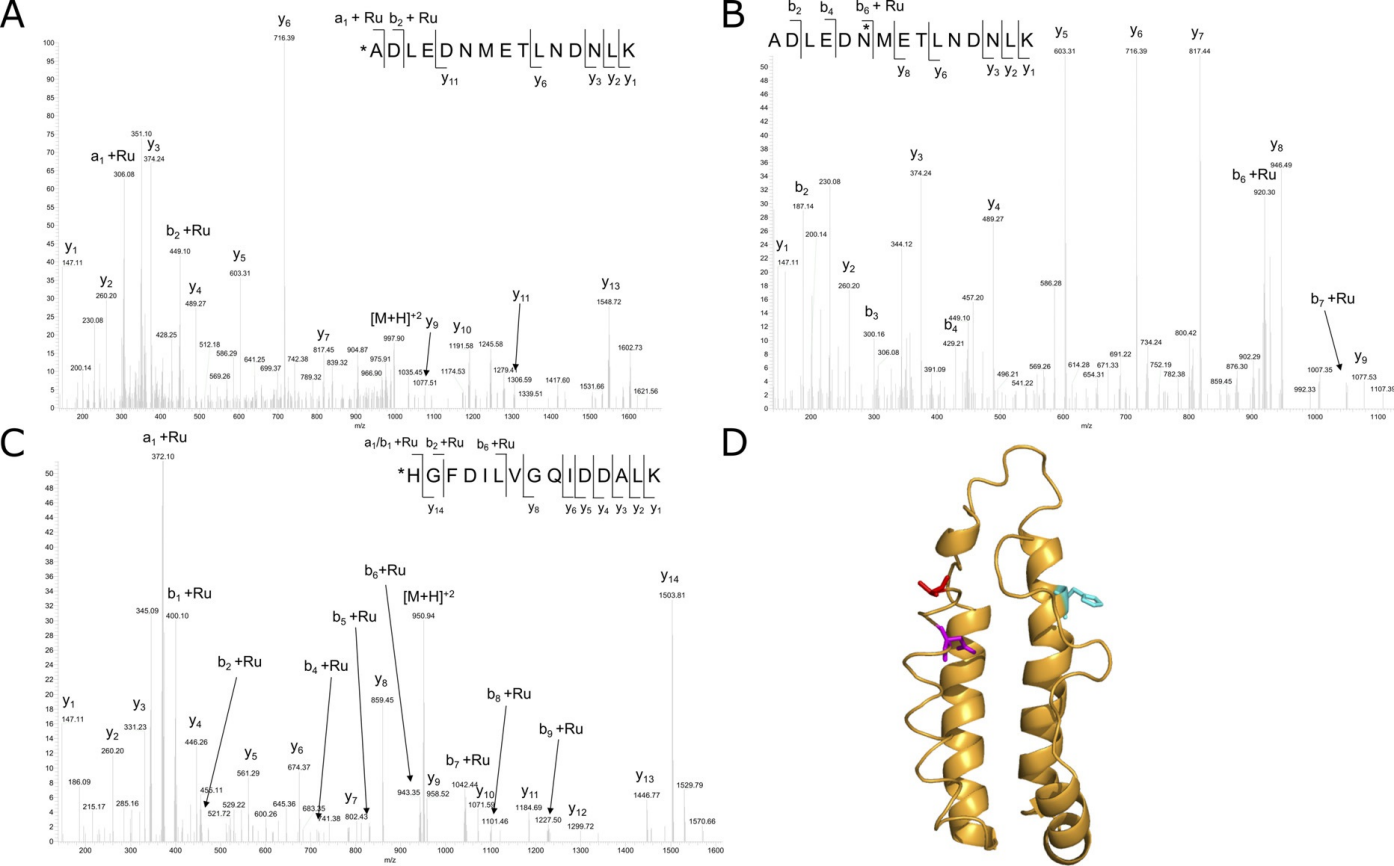
(A–C) LC‐MS/MS analysis of the ruthenium modified peptides 1–15 and 63–77, where Ru corresponds to the [Ru(HMB)] fragment. (A) MS/MS spectra of the peptide **A**DLEDNMETLNDNLK with the a1 + Ru and subsequent ions confirming alanine coordination. (B) MS/MS spectra of the peptide ADLED**N**METLNDNLK with the b6 + Ru ion confirming asparagine coordination. (C) MS/MS spectra of the peptide **H**GFDILVGQIDDALK, with the a1 + Ru, b1 + Ru and subsequent ions confirming histidine coordination. The modified amino acids are highlighted and correspond to the amino acids + a [Ru(HMB)] fragment. (D) A previously reported NMR structure of apocytochrome *b*_562_ with the residues for ruthenium coordination highlighted, N‐terminal alanine (red), Asn6 (magenta) and His63 (cyan).^[68]^

The origin of the observed rate increase can be attributed to the new ligand environment in the activated complex, although tighter substrate binding, non‐covalent protein interactions stabilising the transition state or a combination of all of these could also contribute. To identify the new ligands comprising the first coordination sphere of active ArMs (Hybrids 1–4), we performed tandem MS/MS. This revealed that in all cases the ruthenium was bound by the same amino acids, namely the N‐terminal Alanine and Asn6 on α‐helix 1 and His63 on α‐helix 3, Figure 6. The metal is therefore held between two helices positioned diagonally in the apoprotein, implying that the active complex is embedded within the helical bundle and not at the surface. The coordinating residues must have changed their orientation compared to the average NMR apoprotein structure.^[68]^ Besides inducing rearrangement, association of the metal complex may be favoured for a certain subset of the interchanging apoprotein conformations,[^59^, ^69^] thus potentially explaining the generation of multiple holoprotein isomers following ligand exchange, as seen with the hybrid pairs 1/3 & 2/4. Two of the peptidic ligands, the N‐terminal amino group and the amide group of Asn6 are likely to be weakly coordinating, yet the complexes are stable enough to be detected by mass spectrometry. Nevertheless, these weakly coordinating ligands would potentially lead to faster ligand exchange facilitating transfer hydrogenation activity. Further structural studies are ongoing to elucidate if the complex is distorted from the ideal piano‐stool, pseudo‐octahedral geometry.

Circular dichroism spectra of all these hybrids were very similar to that of the wild‐type protein (Figure S7). Thermal stability measurements of these proteins showed that Hybrids 2 & 4 had a *T*_m_ for denaturation close to the wild type protein but the melting temperature of Hybrids 1 & 3 was raised significantly in comparison, Figure S8. By comparing mass spectrometry data, melting temperature and the transfer hydrogenation activities it can be confidently concluded that Hybrids 1 & 3 are the same species, as are Hybrids 2 & 4. Thus, the same products can be obtained by incubation of one apoprotein with different small molecule complexes, [5] & [6] respectively. The differences between Hybrids 1 & 3 and Hybrids 2 & 4 must result from different topologies around the metal‐protein interactions, possibly different chirality at the metal centre.[^70^, ^71^] The higher melting point together with the observation that more folded proteins tend to produce ion series of lower charge state distribution in the mass spectrometer indicates that the protein scaffold has adopted a fold with more extensive intramolecular bonding in Hybrids 1 & 3 than in Hybrids 2 & 4.^[72]^

## Conclusion

By exploiting the enhanced lability of ruthenium arene bipyridyl complexes in a protein context, protein derived coordination bonds can replace an otherwise inert chelating ligand. This observation allows us to report a straightforward and reliable incubation protocol for the generation of some novel artificial metalloproteins. These functional conjugates contained cofactors displaying multiple protein‐metal coordination bonds; the protein therefore exerts a direct, first‐sphere influence on the metal. Specifically, the products of the reaction of [Ru^II^(η^6^‐arene)(Bipy)Cl]^+^ complexes with Cyt *b*_562_ wt, yielded artificial metalloenzymes capable of catalysing the reduction of a quinolone substrate **1** via transfer hydrogenation from formate. Using a naive protein scaffold, a 35‐fold rate increase was achieved, when compared to the small‐molecule complex in water. Further, this ArM displayed a rate of reaction in water approximately an order of magnitude higher than the known small‐molecule catalyst [Ru(Cym)Cl_2_]_2_ alone. In the future it may be possible to leverage the interdependence afforded by metal‐protein coordination bonds for further optimization by directed evolution, to aim at catalytic rates eventually matching and rivalling the vast field of small‐molecule hydrogenation catalysts but under more benign aqueous conditions and with the high specificity intrinsic to enzymes.[^13^, ^28^, ^29^, ^66^]

A key feature of the method of metal‐protein conjugation presented in this report is that, as a consequence of ligand exchange, the first coordination spheres of the organometallic species in the ArM and the small molecule precursor are distinct in both geometry and ligand identity and therefore chemical properties, including further ligand lability. In particular, ligand exchange upon protein binding allows for using synthetic organometallic complexes that are stable and unreactive in aqueous solution as precursors to active ArMs. The desired catalytic activity can then be unmasked in situ by subsequent reaction with a protein. This has clear advantages for implementing ArMs in living systems, as it reduces potential background activity from the small molecule and allows for protection of the metal centre from the manifold of catalyst poisons present in cells.^[67]^ Furthermore, this strategy still enables the metal centre to carry non‐natural ligands into the enzyme which, besides from activating the metal centre, provides extended functionality that can be recognised by the protein. Such a system has parallels with the apparent mechanism in naturally evolved metalloenzymes. For example, vitamin B12 is catalytically inactive in solution, yet undergoes ligand exchange upon binding to a specific apoprotein, unmasking activity.[^73^, ^74^] In contrast to ArMs where no ligand exchange occurs, reactivity is not ‘imported“ based on an intrinsic property of the metal complex and brought to bear in a protein environment, but *generated* as part of the ArM formation which proceeds via the unprecedented exchange of a bidentate ligand to bring about a reactive complex. Of the few ArM studies that have been reported that make use of a strategy where ligand exchange is central to formation of the artificial holoprotein, to the best of our knowledge, none make use of the potential benefits listed above, as they involve either toxic, water‐sensitive or already very active precursor molecules.[^45^, ^46^, ^47^, ^48^]

With multiple direct coordination bonds between the metal and the protein scaffold, these ArMs can be expected to evolve like their natural counterparts, as protein structure will directly impact the metal properties. Small molecule metal complexes in solution will spontaneously adopt the nearest accessible lowest energy geometry, assuming the ligands can move freely. However, if one or more of the ligands are constituted by the protein, these ligands cannot freely arrange, as they are an integral part of the peptide macromolecule and thus linked cooperatively. A protein scaffold can therefore distort the metal complex to an (in terms of the metal) energetically less favourable geometry, potentially placing the metal into an activated or entatic state.^[75]^ This distortion is possible by compensating the energetic cost of strain and low metal coordination energy with the many other non‐covalent bonding interactions that form the three‐dimensional structure of the protein. Thus, when subjugating the protein to evolutionary pressures, the core metal complex can constitute part of the evolutionary response and therefore an influence can be exerted over the fundamental chemically transforming step. When entire metal complexes are conjugated to protein hosts, as often found in supramolecular and covalent methods of generating ArMs, this is not possible, and may be a factor limiting maximum activity in previous directed evolution campaigns.[^19^, ^27^, ^28^] With ArMs promising to play a significant role in achieving greener catalytic chemistry, overcoming such limits using simple and easily available methodology will be key in fulfilling these expectations.

## Conflict of interest

The authors declare no conflict of interest.

## Supporting information

As a service to our authors and readers, this journal provides supporting information supplied by the authors. Such materials are peer reviewed and may be re‐organized for online delivery, but are not copy‐edited or typeset. Technical support issues arising from supporting information (other than missing files) should be addressed to the authors.

Supplementary
