# Supplementary material for: Controlled Ligand Exchange Between Ruthenium Organometallic Cofactor Precursors and a Naïve Protein Scaffold Generates Artificial Metalloenzymes Catalysing Transfer Hydrogenation
Source: Angew Chem Int Ed Engl. 2021 Mar 26;60(19):10919–27. doi: 10.1002/anie.202015834 (PMC8251807; doi:10.1002/anie.202015834)
Supplement: Supplementary file 1 — Supplementary [file ANIE-60-10919-s001.pdf]

Angewandte Chemie
Eine Zeitschrift der Gesellschaft Deutscher Chemiker

Supporting Information

Controlled Ligand Exchange Between Ruthenium Organometallic Cofactor Precursors and a Naïve Protein Scaffold Generates Artificial Metalloenzymes Catalysing Transfer Hydrogenation

George S. Biggs[+], Oskar James Klein[+], Sarah L. Maslen, J. Mark Skehel, Trevor J. Rutherford, Stefan M. V. Freund, Florian Hollfelder, Sally R. Boss, and Paul D. Barker**

anie_202015834_sm_miscellaneous_information.pdf

Table of Contents

General Experimental Details 2
Supplementary Figures 8
Chemical Synthesis and Characterisation 16
References 34

General Experimental Details

Liquid Chromatography Mass Spectrometry

Protein LC–MS was performed on a Xevo G2-S TOF mass spectrometer coupled to an Acquity UPLC system using an Acquity UPLC BEH300 C4 column (1.7 µm, 2.1 × 50 mm). H_2O with 0.1 % formic acid (solvent A) and 95 % acetonitrile and 5 % water with 0.1 % formic acid (solvent B), were used as the mobile phase at a flow rate of 0.2 mL/min. The gradient was programmed as follows: 95% A for 0.93 min, then a gradient to 100% B over 4.28 min, then 100% B for 1.04 minutes, then a gradient to 95% A over 1.04 min. The electrospray source was operated with a capillary voltage of 2.0 kV and a cone voltage of 40 V. Nitrogen was used as the desolvation gas at a total flow of 850 L/h. Total mass spectra were reconstructed from the ion series using the MaxEnt algorithm preinstalled on MassLynx software (v4.1 from Waters) according to the manufacturer's instructions.

Nuclear Magnetic Resonance

All NMR data were collected at 298 K (unless stated) using Bruker Avance spectrometers with ^{1}H resonance frequencies of 400, 500, 600 MHz.

For proton NMR, chemical shifts (δH) are reported in parts per million (ppm), to the nearest 0.01 ppm and are referenced to the residual non-deuterated solvent peak. Coupling constants (*J*) are reported in Hertz (Hz) to the nearest 0.1 Hz. Data are reported in the order: (i) chemical shift, (ii) multiplicity (s = singlet; d = doublet; t = triplet; q = quartet; sep = septet; m = multiplet; or as a combination of these, e.g. dd, dt etc.), (iii) coupling constant(s), (iv) integration and (v) assignment. For carbon NMR, chemical shifts (δC) are quoted in ppm, to the nearest 0.1 ppm, and are referenced to the residual non-deuterated solvent peak. ^{19}F (470 MHz) chemical shifts were referenced to the unified scale according to IUPAC recommendations, using the ^{1}H signal of external dimethylsilapentanesulfonate (DSS) in H_2O as the reference standard.

Circular Dichroism

CD spectra were recorded in 1 mm pathlength quartz cuvettes using a Chirascan CD spectrometer. CD data were recorded from 300 – 185 nm with sampling at 0.5 nm intervals. Samples were made to an expected concentration of approximately 5 µM in low buffer concentration.

Temperature denaturation experiments were performed measuring ellipticity (θ) with a fixed λ = 222 nm across a temperature range of 25 – 90 °C with a linear increase of 1 °C / min.

Elemental Analysis

Elemental microanalytical data were obtained from the University of Cambridge, Department of Chemistry microanalytical service. C,H,N analysis was carried out using an Exeter Analytical CE-440 Elemental Analyser by combustion of the sample under a pure O_2 atmosphere at 975 ºC.

ICP-OES analyses were carried out on a Thermo Scientific iCAP 7400 Duo. Double-deionized water was used for all analysis. The Ruthenium Specpure plasma standard (ruthenium chloride, 1004 ± 5 μg/mL in 10% v/v hydrochloric acid) was diluted with HNO_3 to 2% HNO_3 and calibrants were prepared at concentrations of 1 − 1000 ppb. Samples were prepared through dilution in 2% v/v HNO_3.

Plate Reader

All fluorescence spectroscopy measurements in a 96-well plate format were performed on a SpectraMax i3x plate reader (Molecular Devices). Measurements were taken at λ_{Ex} = 370 nm, λ_{Em} = 460 nm, bottom read, PMT = high, 5 s orbital shaking at the start and 1 s between measurements and in 96-well plates. Wells were sealed with tape to prevent evaporation of solution.

Protein Expression and Purification

Ubiquitin K63C

2.5 μL of ubiquitin plasmid #86589 (Addgene) was transformed into 50 μL of super-competent Bl21-Gold(DE3) cells by electroporation. Following this 1 mL of 2 x YT media was added to the cuvette which was incubated at 37 ºC for 45 minutes. 200 μL of cells was spread onto ampicillin plates and incubated at 37 ºC overnight to allow for colonies to grow. A single colony from this culture was transferred to 20 mL of LB media with ampicillin (100 μg/mL) and incubated overnight at 37 ºC and 180 rpm.

This culture was used to inoculate 2 L of LB medium containing ampicillin (100 μg/mL) and cells were grown to OD600 of 0.6 (37 ºC, 180 rpm). Expression of ubiquitin K63C was induced with 1mM IPTG and protein was expressed at 18 ºC overnight. The cells were then harvested by centrifugation at 4 ºC and 4000 g for 30 minutes. Supernatant was discarded and cells were

resuspended in 50 mM NaPi buffer (pH 7.4) containing 250 mM of NaCl and, 25 mM imidazole and 1 mM TCEP and a protease inhibitor cocktail. Cells were lysed by sonication and cell fragments were collected and removed by centrifugation. The supernatant was purified by IMAC chromatography with a linear gradient of imidazole from 25 mM to 500 mM. Samples containing ubiquitin K63C were combined and cleavage of the fusion partner maltose binding protein (MBP) was conducted by incubating a 1:20 molar ratio of TEV protease at 25 °C 24 hr. Following cleavage, the protein was purified by IMAC chromatography to remove MBP and TEV protease. Further purification was often required (confirmed by SDS-gel electrophoreses) and this was performed through a Mono S cation exchange (high performance) in 20 mM MES buffer pH = 5.0 developed with a linear gradient of 0 – 200 mM KC1. The flow-through was pooled, concentrated and buffer exchange to 50 mM NaPi buffer pH 8 was performed.

Sequence

SAQIFVKTLTGKTITLEVEPSDTIENVKAKIQDKEGIPPDQQRLIFAGKQLEDGRTLSD
YNIQCESTLHLVLRLRGG

M_w – 8567 Da

Cytochrome b_{562}

1 - 2 μL of the required cytochrome plasmid[1,2] was transformed into 50 μL of super-competent Bl21-Gold(DE3) cells by electroporation. Following this 1 mL of 2 x YT media was added to the cuvette which was incubated at 37 ºC for 45 minutes. 200 μL of cells was spread onto ampicillin resistant plates and incubated at 37 ºC overnight to allow for colonies to grow. A single colony from this culture was transferred to 20 mL of 2 x YT media with ampicillin (100 μg/mL) and incubated overnight at 37 ºC and 180 rpm to give a pre-culture.

10 mL of the pre-culture was inoculated into a 1 L culture 2 x YT media with ampicillin (100 μg/mL) in a 2 L flask and this was shaken at 200 rpm at 37 ºC, conditions considered to be microaerobic. Innoculae were grown to an optical density at 600 nm of about 0.6 before induction with 0.1 mM IPTG, and the cultures continued into stationary phase before harvest by centrifugation at 6 hr after induction. The cells were then harvested by centrifugation at 4 ºC and 4000 g for 30 minutes. Following cell growth, cytochrome b_{562} is found in both the periplasmic fractions and the culture medium, however, for practical reasons only cytochrome b_{562} proteins in the periplasm were harvested, therefore supernatant was discarded.

Protein was harvested from E. coli cells by fractionation of the pelleted cells into spheroplasts and soluble periplasm contents using the lysozyme/EDTA method. The apo- and holo-cytochromes in these fractions were separated on a Q-Sepharose anion exchanger in diethanolamine (DEA, 20 mM, pH 8.5) developed with a linear gradient of 0 – 300 mM KCl. Further purification was often required (confirmed by SDS-gel electrophoreses) and this was performed on a Superdex S75 gel filtration column in 50 mM NaPi buffer with 100 mM NaCl and 1 mM TCEP.

<u>Sequences</u>

ADLEDNMETLNDNLKVIEKADNAAQVKDALTKMRAAALDAQKATPPKLEDKSPDS
PEMKDFRHGFDILVGQIDDALKLANEGKVKEAQAAAEQLKTTRNAYHQKYR

WT M_w – 11780 Da

ADLEDNMET**<u>C</u>**NDNLKVIEKADNAAQVKDALTKMRAAALDAQKATPPKLEDKSPDS
PEMKDFRHGFDILVGQIDDALKLANEGKVKEAQAAAEQLKTTRNAY**<u>M</u>**QKYR

L10C H102M M_w – 11764 Da

Protein Conjugation

To an Eppendorf tube with NaPi (50 mM, pH 8.0) and DMF (10% of total volume), an aliquot of a stock solution of protein (final concentration either 10 µM, 50 µM or 200 µM) was added. Afterwards, a solution of the ruthenium complex (1 to 20 equiv.) in DMF was added and the resulting mixture was vortexed for 10 seconds. The reaction was mixed for up to 24 h, at 37 °C. At desired time points, an aliquot was taken and diluted to a final concentration of 5 µM with water and analysed by LC–MS.

For modifications with N-ethyl maleimide, from a stock 10mM solution in DMF, 1 Eq. of reactant was added and the mixture vortexed for 10 seconds, followed by incubation for 30 mins at 37 °C. Modification was confirmed by LC-MS.

Hybrid Purification and Quantification

Upon modification protein – ruthenium hybrids were purified by anion exchange chromatography. The incubation was buffer exchanged into DEA (20 mM, pH 8.5), thus removing some of the excess ruthenium complex, and concentrated to the required volume for anion exchange. The anion exchange was performed with a linear gradient of 0 – 200 mM KCl. and protein components analysed by LC–MS.

Desired fractions were combined, buffer exchanged into water and concentrated to 1 mL. 250 µL of this was diluted to 10 mL in 2 % HNO_3 and analysed for ruthenium concentration *via* ICP-OES.

Tandem MS/MS Analysis

Protein samples (30µM) were prepared for mass spectrometric analysis by hand, solution samples were digested with trypsin (Promega, UK) at a 1:50 ratio overnight at 37 °C. The resulting peptides were diluted in 2% v/v formic acid, 2% v/v acetonitrile. The digests were analysed by nano-scale capillary LC-MS/MS using an Ultimate U3000 HPLC (ThermoScientific Dionex, San Jose, USA) to deliver a flow of approximately 300 nL/min. A C18 Acclaim PepMap100 5 µm, 100 µm x 20 mm nanoViper (ThermoScientific Dionex, San Jose, USA), trapped the peptides prior to separation on a C18 T3 1.8 µm, 75 µm x 250 mm analytical UPLC column (Waters, UK). Peptides were eluted with a 30 minute gradient of acetonitrile (2% to 40%). The analytical column outlet was directly interfaced *via* a nano-flow electrospray ionisation source, with a quadrupole Orbitrap mass spectrometer (Q-Exactive HFX, ThermoScientific, USA). MS data were acquired in data-dependent mode using a top 10 method, where ions with a precursor charge state of +1 were excluded. High-resolution full scans (R = 60,000, m/z 300-1800) were recorded in the Orbitrap followed by higher energy collision dissociation (HCD) (26 % Normalized Collision Energy) of the 10 most intense MS peaks. The fragment ion spectra were acquired at a resolution of 15 000 and dynamic exclusion window of 20 s was applied.
LC-MS/MS data were then searched against an in-house database using the Mascot search engine programme (Matrix Science, UK). Database search parameters were set with a precursor tolerance of 30 ppm and a fragment ion mass tolerance of 0.1 Da. One missed enzyme cleavage was allowed and variable modifications for ruthenium fragments allowed on the protein N-terminus, Cys, His, Lys, Asn, Gln, Arg and Trp were included. MS/MS data were validated using the Scaffold programme (Proteome Software Inc., USA).[3] All data were additionally interrogated manually.

Transfer Hydrogenation Assay

Ruthenium Dimers and Complexes

Ruthenium compounds were dissolved in DMF to yield 5 mM stock solutions. These were diluted 1:5 with water fresh before each experiment. 80 µL of water, 10 µL of ruthenium solution, 10 µL of buffer (500 mM sodium phosphate, 1 M formate which upon 1 in 10 dilution

gave a pH 8.0) and 1 µL substrate **1** stock solution (100 mM in DMSO) were pipetted into a clear-bottom 96 well plate (final concentrations 100 µM ruthenium compound, 100 mM formate, 1 mM substrate **1**). The wells were sealed with tape and the fluorescence measured over 16 h. Typical time courses are shown in **Fig. S3**.

Protein-Ruthenium Hybrids

90 µL of each protein sample (in water) of known concentration was pipetted into a clear-bottom 96 well plate. 10 µL of buffer (500 mM sodium phosphate, 1 M formate which upon 1 in 10 dilution gave a pH 8.0) and 1 µL of substrate **1** stock solution (100 mM in DMSO) was added to each well (final concentrations 100 mM formate, 1 mM substrate **1**). The wells were sealed with tape and the fluorescence was measured over 16 h. Typical time courses are shown in **Fig. S4**.

Supplementary Figures

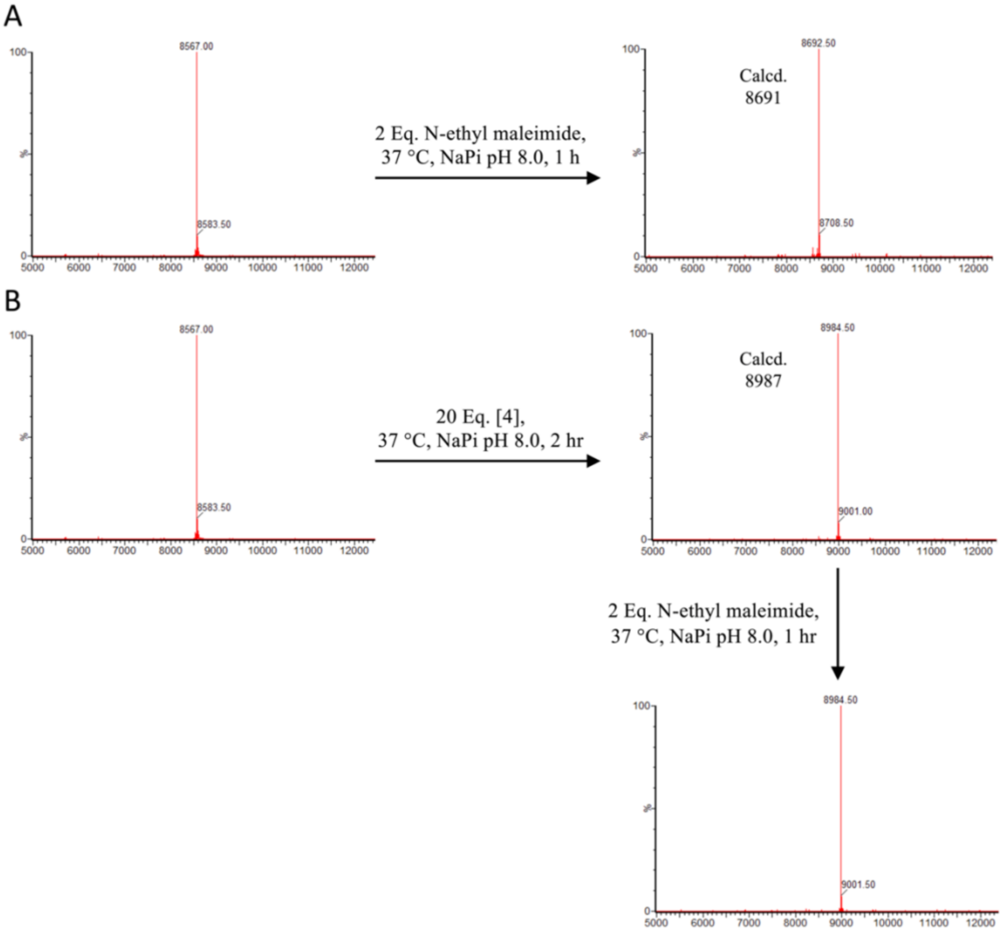

Figure S1: (A) Modification of Ubq K63C with the small cysteine modifying molecule N-ethyl maleimide (50 μM protein). **(B)** Modification of Ubq K63C with complex [4] and subsequent lack of modification with N-ethyl maleimide (50 μM protein). This confirms that the site of initial ruthenium coordination for Ubq K63C is the cysteine residue.

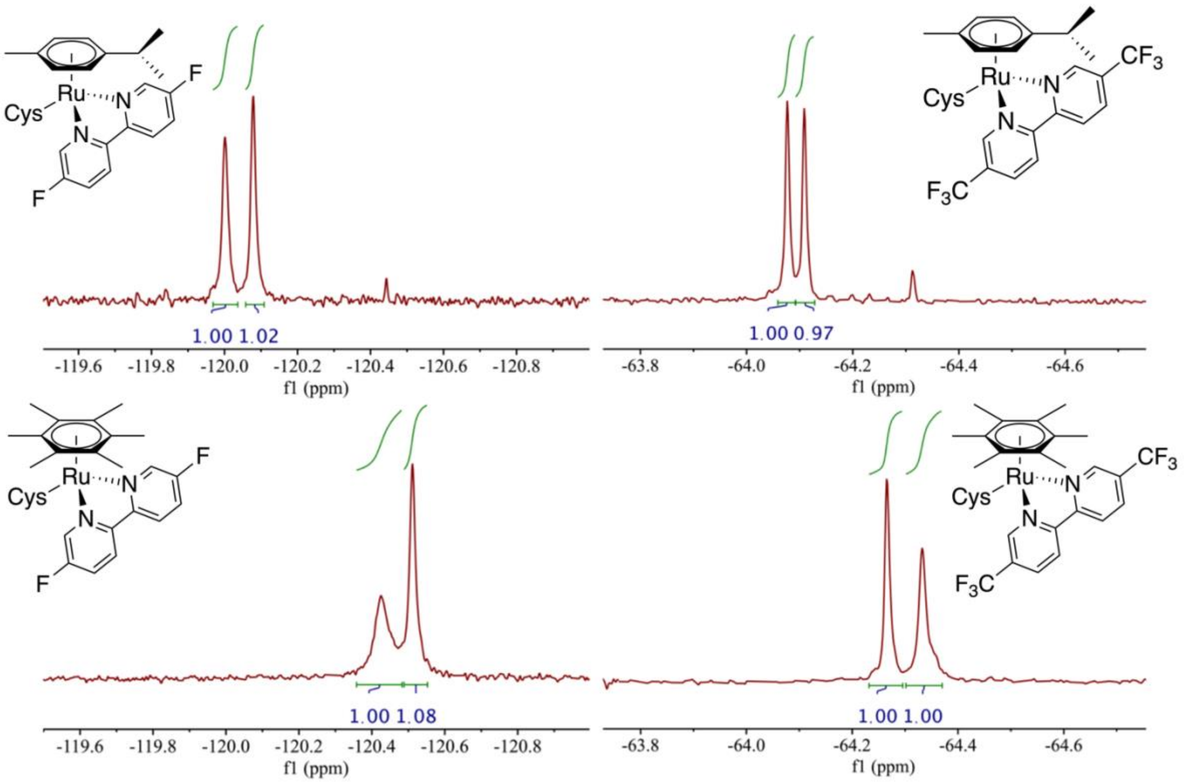

Figure S2: 470 MHz $^{19}F\{^{1}H\}$ NMR spectra of four different Ru-Cyt b_{562} Cys adducts. Adducts were placed in 90% Buffer: 10% D_2O and a 256 scan experiment was performed with 0.7 s recycle time and 0.7 Hz/point digital resolution. The respective ruthenium cofactors are shown on each spectra.

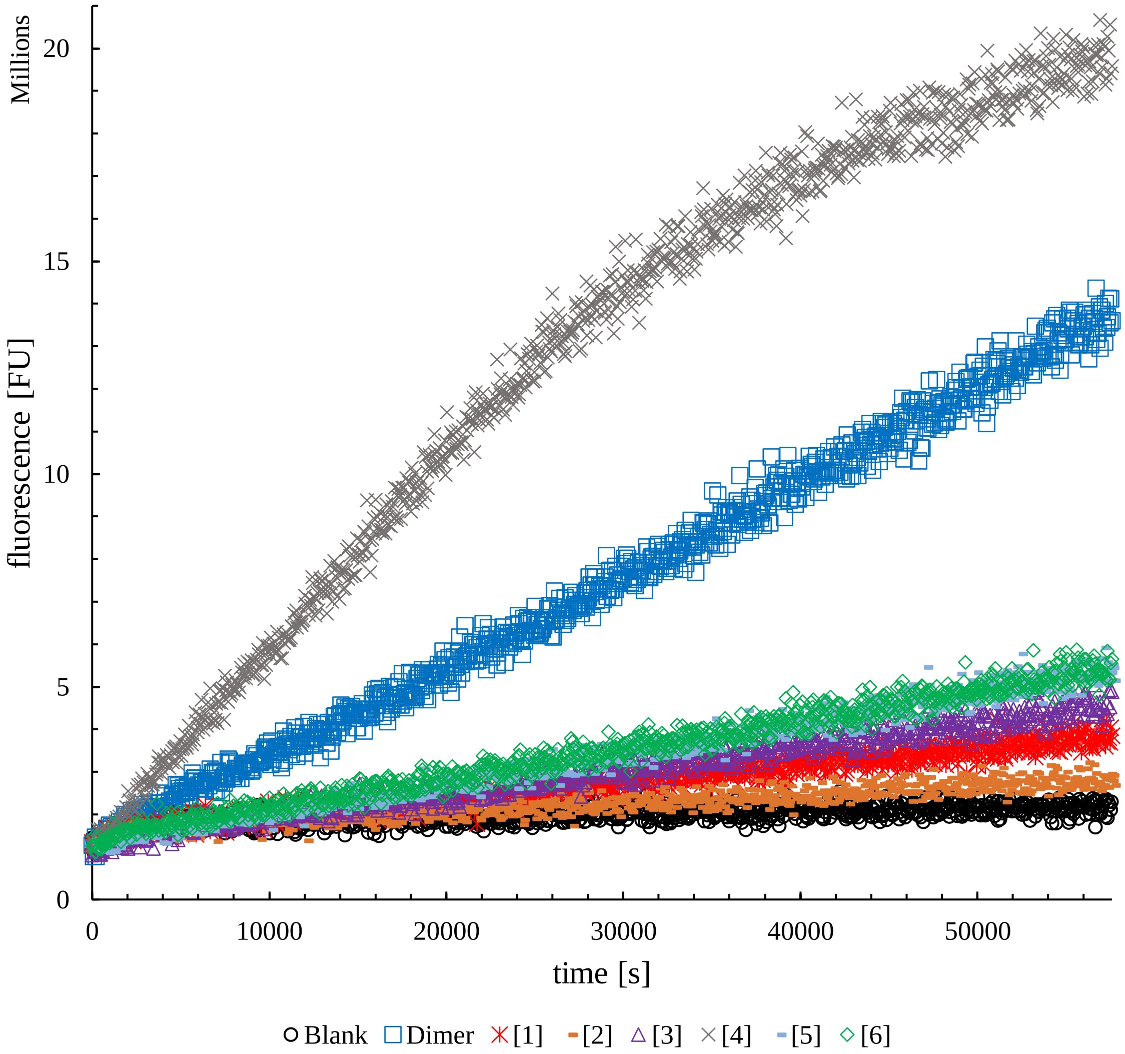

Figure S3: Typical time courses obtained for assaying transfer hydrogenation activity of the small molecule complexes. *Conditions*: 100 µM ruthenium compound, 50 mM sodium phosphate, pH 8.0, 100 mM formate, 1 mM substrate **1**, measured over 16 h at 37 °C

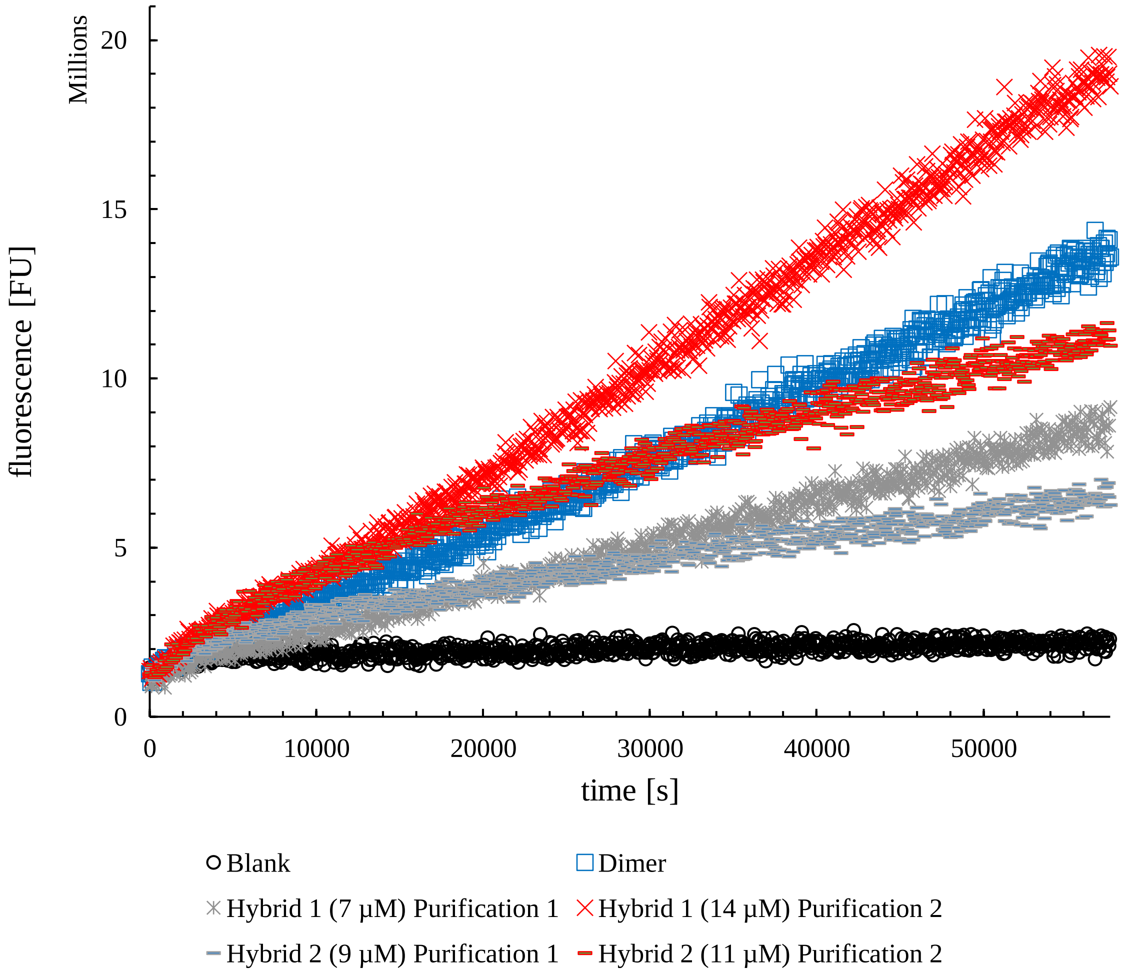

Figure S4: Typical time courses obtained for assaying transfer hydrogenation activity of the protein-ruthenium hybrids. Concentrations of hybrids were determined *via* ICP-OES. *Conditions*: 50 mM sodium phosphate, pH 8.0, 100 mM formate, 1 mM substrate **1**, measured over 16 h at 37 °C

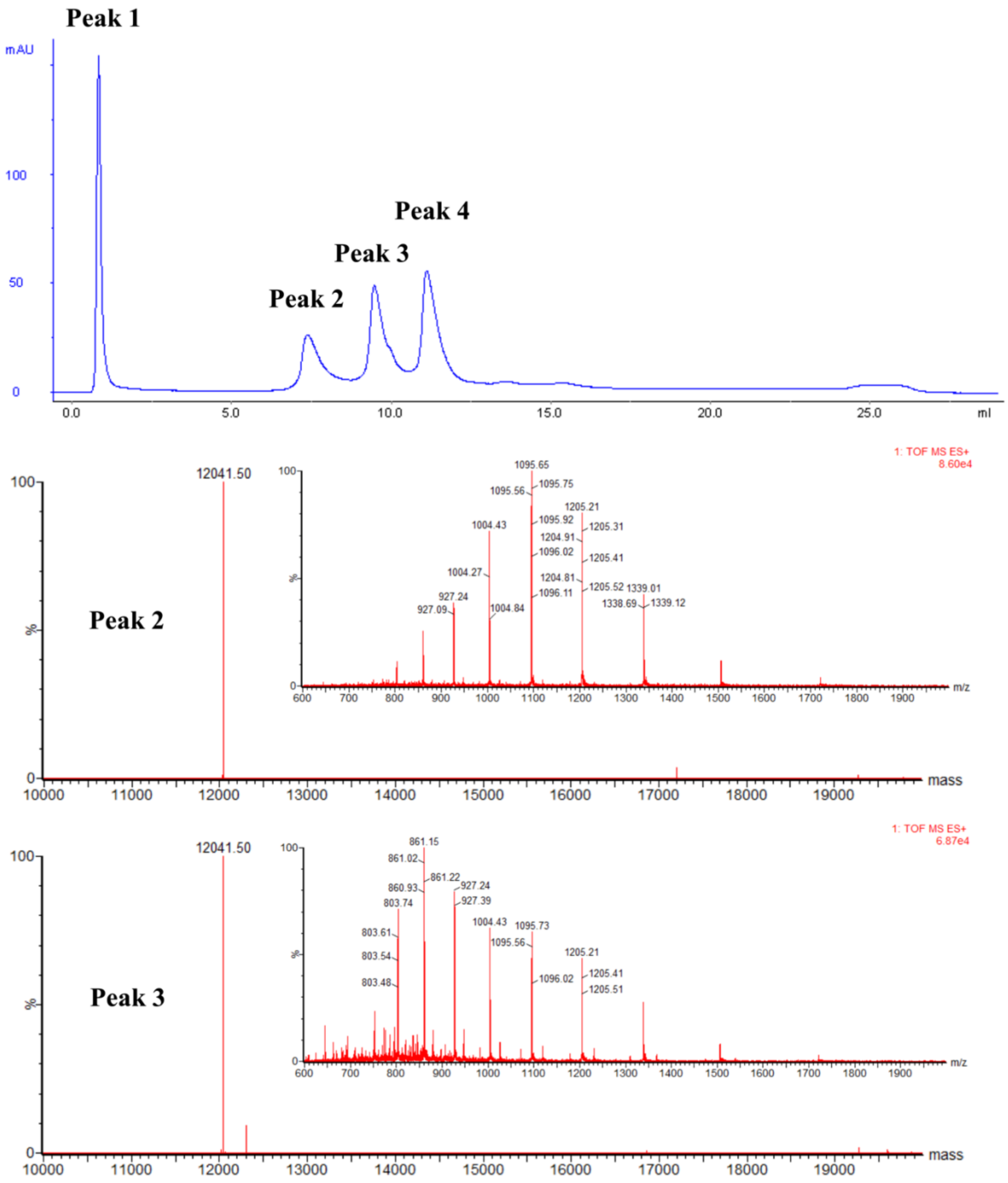

Figure S5: (Top) UV trace from an anion exchange purification from an incubation of complex [6] with Cyt *b*562 wt. Peak 1 corresponds to unreacted ruthenium complex and peak 4 corresponds to unreacted Cyt *b*562 wt. (**Middle and Bottom):** Deconvoluted mass spectra and ion series (insert) of the different modified protein species that come off the ion exchange column.

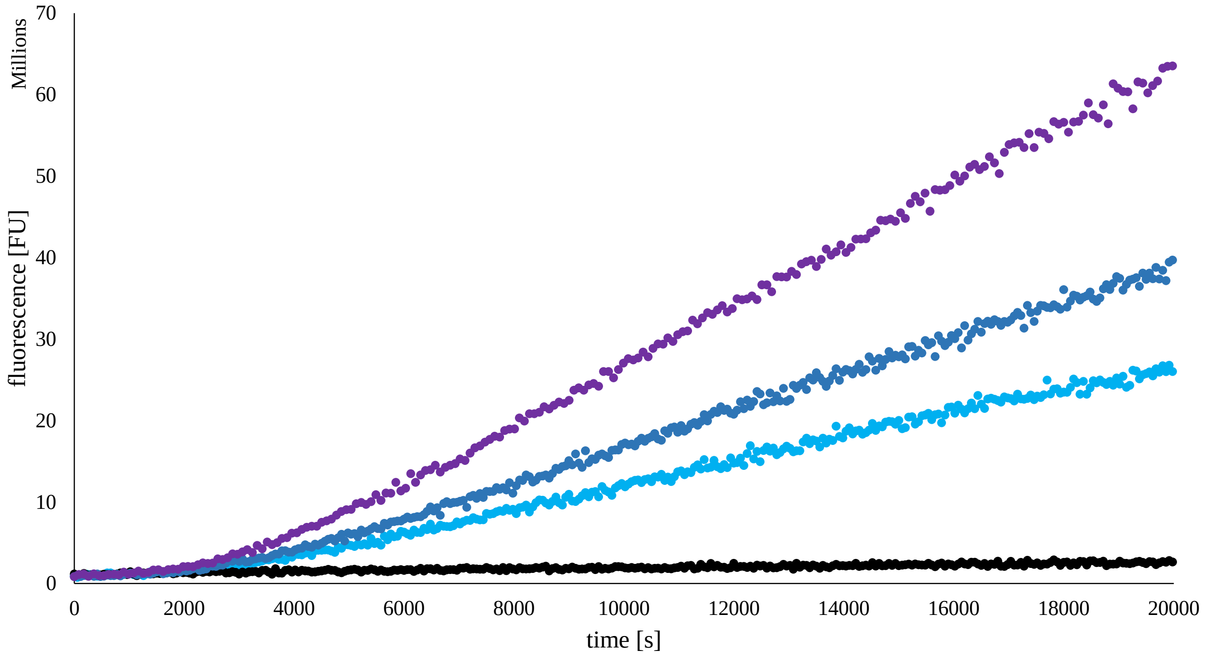

Figure S6: A graph showing the evolution of fluorescence activity upon hybrid formation in comparison to free complex [6] (black trace). The transfer hydrogenation substrate is combined with Cyt b_{562} wt and complex [6] at the start of the experiment. Varying equivalents of complex [6] are added 0.5 Eq. (light blue trace), 1 Eq. (dark blue trace) and 2 Eq. (purple trace). The increased rate observed with 2 Eq. of complex [6] we attribute to the formation of an active doubly modified hybrid, which has been confirmed in the LC-MS. *Conditions*: 100 µM protein, 50 mM sodium phosphate, pH 8.0, 100 mM formate, 1 mM substrate **1**, measured over 16 h at 37 °C

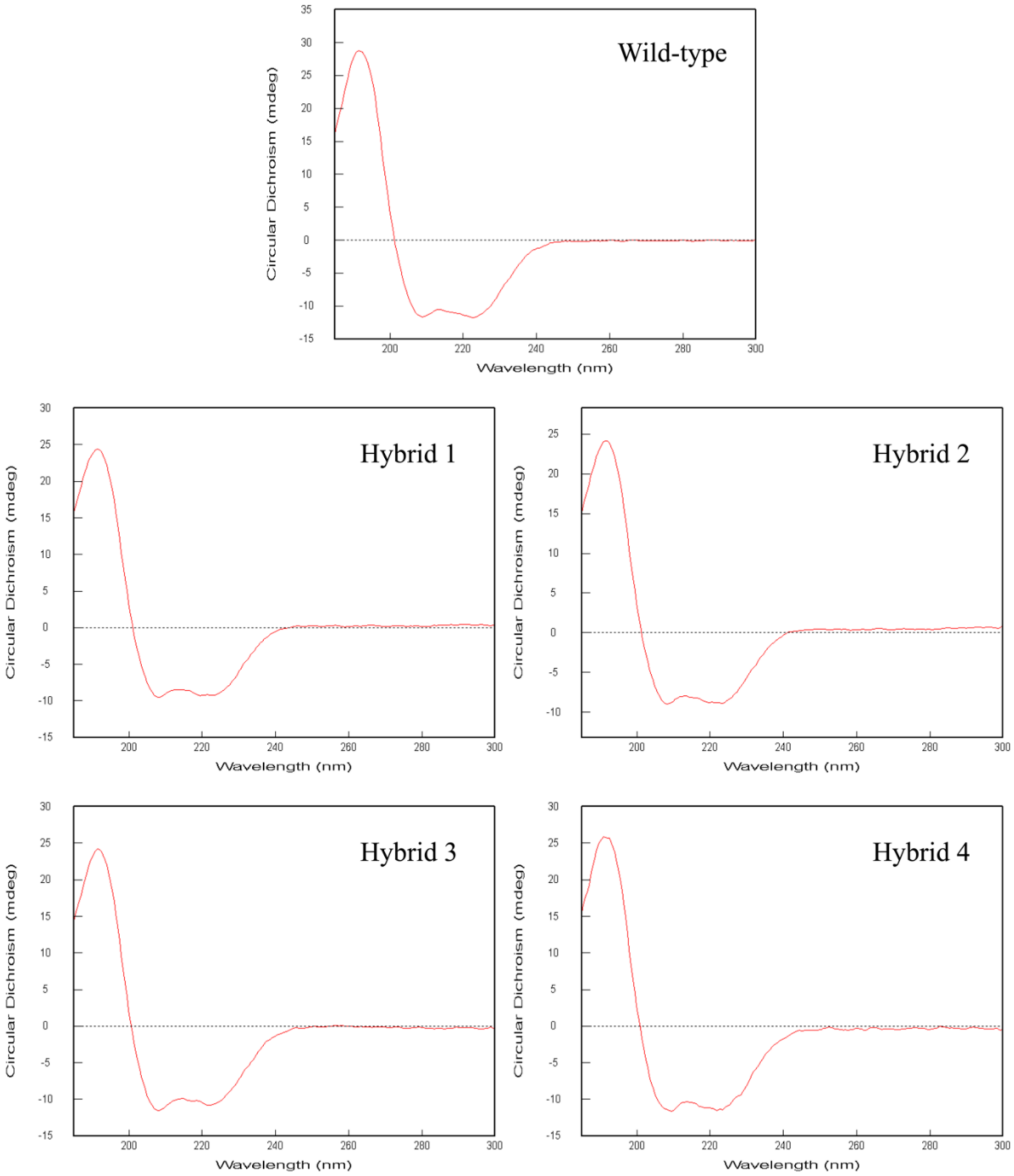

Figure S7: CD spectra of the Cyt *b*$_{562}$ wt and Hybrids 1 - 4 (5 µM protein concentration, 10 mM sodium phosphate buffer pH 8.0, recorded at 25 °C). Concentrations of hybrids were determined *via* ICP-OES.

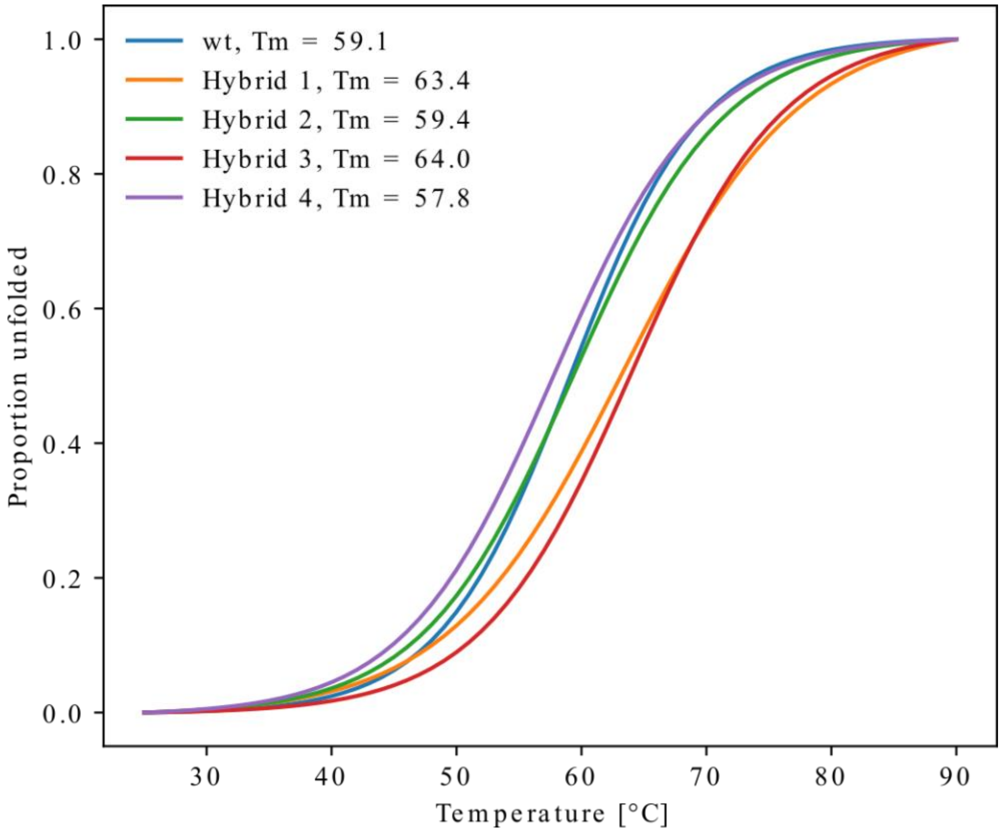

Figure S8: Normalised measure of proportion of unfolded protein as a function of temperature for Cyt b_{562} wt and Hybrids 1-4. The melting temperatures are given for the five different protein species (5 μM protein concentration, 10 mM sodium phosphate buffer pH 8.0, recorded between 25 °C and 90 °C with a increase of 1 °C / minute). Concentrations of hybrids were determined *via* ICP-OES.

Chemical Synthesis and Characterisation

General synthesis of fluorinated bipyridines (adapted from the procedure of Lee *et al.*[4])

The starting bromopyridine (1.0 mmol), $Pd(OAc)_2$ (0.025 mmol), indium (0.5 mmol) and lithium chloride (1.5 mmol) in dry DMF (2 mL) were stirred at 100 °C for 1 h under a nitrogen atmosphere. The reaction mixture was quenched with $NaHCO_3$ (saturated aqueous). The aqueous layer was extracted with ethyl acetate (3 x 20 mL) and the combined organic phase was washed with water (20 mL) and brine (20 mL), dried with $MgSO_4$, and filtered. The residue was purified by silica gel column chromatography (Ethyl acetate:hexane = 1:2).

General synthesis of Ru(II)(η^6-arene)(bipyridine) complexes [1] – [6]

Either [Ru(*p*-cymene)Cl_2]$_2$ or [Ru(hexamethylbenzene)Cl_2]$_2$ (0.23 mmol) and appropriate bipyridine (0.47 mmol) were added to a nitrogen purged flask. Freshly distilled MeOH (25 mL) was added and the reaction was stirred for 24 hours at room temperature. The contents were filtered and the solution was reduced to approximately 5mL *in vacuo.* NH_4PF_6 (230 mg, 1.40 mmol) was added and the mixture was shaken and left at -10 °C for a further 24 hours. The product was collected by gravity filtration.

Synthesis of the transfer hydrogenation substrate 1,2-dimethyl-6-(((2-oxo-2H-chromen-7-yl)oxy)methyl)quinolin-1-ium – 1 (adapted from the procedure of Ward *et al.*[5])

(2-methyl-6-quinolinyl)methanol (100 mg, 0.58 mmol, 1 Eq.), methanesulfonic anhydride (110 mg, 0.63 mmol, 1.1 Eq.) and DMAP (7.3 mg, 0.06 mmol, 0.1 Eq.) were weighed into a dry Schlenk tube and dried under vacuum for 1 h. The system was put under a N_2 atmosphere and CH_2Cl_2 (5 mL), CH_3CN (1 mL) and anhydrous pyridine (47 μL, 0.58 mmol. 1 eq.) were added. The mixture was stirred at room temperature for 30 min, after which the solvent was removed *in vacuo*. Umbelliferone (113 mg, 0.70 mmol, 1.2 eq.) and caesium carbonate (331 mg, 1.02 mmol, 1.75 eq.) were dried *in vacuo* before addition of CH_3CN (10 mL). After stirring for 20 min., the mesylated quinoline was taken up in CH_3CN (10 mL) and small portions were added slowly to the umbelliferone mixture. After complete addition, the mixture was stirred at room temperature overnight. The mixture was filtered, the solvent was removed *in vacuo* and the residue taken up in EtOAc (20mL). The mixture was washed with 1N $NaOH_{(aq)}$ (3 x 20 mL), after which the combined aqueous phase was extracted with EtOAc (2 x 20 mL). The product

(142 mg, 0.45 mmol, 77% yield) was obtained as a white solid after flash chromatography (EtOAc:Hex 2:1).

The coupled product (132 mg, 0.42 mmol, 1 eq.) was dissolved in dry toluene (30 mL) under a N_2 atmosphere. Dimethyl sulphate (395 μL, 4.2 mmol, 10 eq.) was added and the mixture stirred for 24 h at 100 °C. The product was obtained by filtration and washing with Et_2O and hexane as an off-white solid (57 mg, 0.13 mmol, 31% yield).

Characterisation of transfer hydrogenation substrate – 1

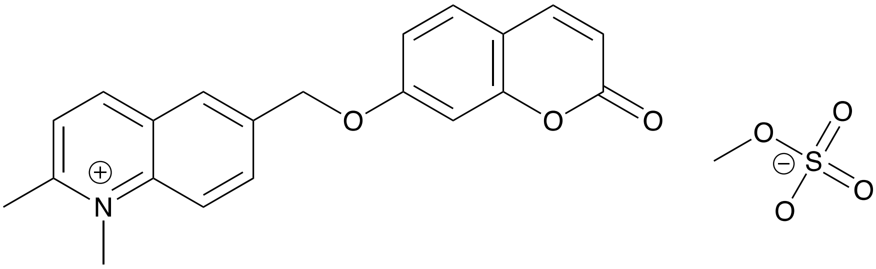

^{1}H NMR (400.13 MHz, d^{6}-DMSO): δ (ppm) 9.11 (d, $^{3}J_{HH}$ = 8.6 Hz, 1H), 8.64 (d, $^{3}J_{HH}$ = 9.2 Hz, 1H), 8.47 (s, 1H), 8.29 (dd, $^{3}J_{HH}$ = 9.2, $^{4}J_{HH}$ = 1.9 Hz ,1H), 8.13 (d, $^{3}J_{HH}$ = 8.6 Hz, 1H), 8.02 (d, $^{3}J_{HH}$ = 9.5 Hz, 1H), 7.69 (d, $^{3}J_{HH}$ = 8.6 Hz, 1H), 7.16 (d, $^{4}J_{HH}$ = 2.2 Hz, 1H), 7.11 (dd, $^{3}J_{HH}$ = 8.6, $^{4}J_{HH}$ = 2.4 Hz, 1H), 6.33 (d, $^{3}J_{HH}$ = 9.5 Hz, 1H), 5.57 (m, 2H), 4.45 (s, 3H), 3.37 (, 3H) 3.08 (s, 3H).

Characterisation of 5,5'-difluorobipyridine – 2

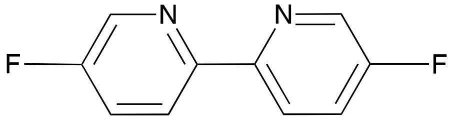

Synthesised with the starting material 2-bromo-5-fluoropyridine. Yield: 78%. Appearance: White solid

^{1}H NMR (400.13 MHz, $CDCl_3$): δ (ppm) 8.50 (d, $^{4}J_{HH}$ = 2.8 Hz, 2H, 6,6'-position), 8.38 (dd, $^{3}J_{HH}$ = 8.7 Hz, $^{4}J_{HF}$ = 4.5 Hz, 2H, 3,3'-position), 7.52 (dd, $^{3}J_{HH}$ = 8.7 Hz, $^{4}J_{HH}$ = 2.8 Hz, 2H, 4,4'-position). **^{13}C{^{1}H} NMR** (100.57 MHz, $CDCl_3$): δ (ppm) 160.0 (d, $^{1}J_{CF}$ = 258 Hz, 5,5'-position), 151.7 (d, $^{4}J_{CF}$ = 4 Hz, 2,2'-position), 137.4 (d, $^{2}J_{CF}$ = 24 Hz, 6,6'-position), 123.8 (d, $^{2}J_{CF}$ = 18 Hz, 4,4'-position), 122.3 (d, $^{3}J_{CF}$ = 5 Hz, 3,3'-position). **^{19}F{^{1}H} NMR** (376.50 MHz, $CDCl_3$): δ (ppm) -127.4 (s).

Characterisation of 5,5'-di(trifluorometthy)bipyridine – 3

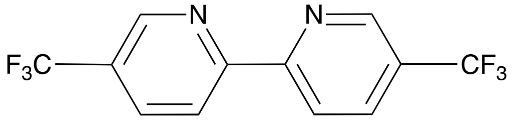

Synthesised with the starting material 2-bromo-5-(trifluoromethyl)pyridine. Yield: 65%. Appearance: White solid

^{1}H NMR (400.13 MHz, $CDCl_3$): δ (ppm) 8.99 (d, $^{4}J_{HH}$ = 1.8 Hz, 2H, 6,6'-position), 8.65 (d, $^{3}J_{HH}$ = 8.4 Hz, 2H, 3,3'-position), 8.12 (dd, $^{3}J_{HH}$ = 8.4 Hz, $^{4}J_{HH}$ = 1.8 Hz, 2H, 4,4'-position).

$^{13}C\{^1H\}$ **NMR** (100.57 MHz, $CDCl_3$): δ (ppm) 157.8 (s, 2,2'-position), 146.5 (q, $^3J_{CF}$ = 3.9 Hz, 6,6'-position), 134.5 (q, $^3J_{CF}$ = 3.9 Hz, 4,4'-position), 127.3 (q, $^2J_{CF}$ = 33 Hz, 5,5'-position), 123.7 (q, $^1J_{CF}$ = 273 Hz, $\underline{C}F_3$,), 121.4 (s, 3,3'-position). $^{19}F\{^1H\}$ **NMR** (376.50 MHz, $CDCl_3$): δ (ppm) -62.4 (s).

Characterisation of [Ru(η⁶-*p*-cymene)(bipyridine)Cl][PF₆] – [1]

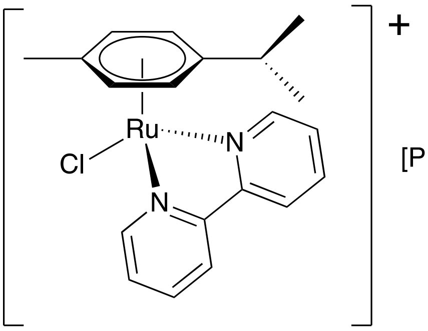

Yield: 64%. Recrystallised via vapour diffusion Et_2O:Acetone. Appearance: Yellow prisms.

Elemental: Anal. Calcd for $C_{20}H_{22}ClF_6N_2PRu$: C, 42.00; H, 3.88; N, 4.90. Found: C, 41.89; H, 3.86; N, 4.78 **HRMS (ESI⁺):** *m/z* 427.0625 $[M – PF_6]^+$ (m_{calc} = 427.0515). **^{1}H NMR** (400.13 MHz, d^6-DMSO): δ (ppm) 9.54 (d, $^3J_{HH}$ = 5.8 Hz, 2H, 6,6'-position), 8.65 (d, $^3J_{HH}$ = 8.0 Hz, 2H, 3,3'-position), 8.29 (overlapping dd, $^3J_{HH}$ = 8.6 Hz, $^3J_{HH}$ = 8.0 Hz, 2H, 4,4'-position), 7.80 (overlapping dd, $^3J_{HH}$ = 8.0 Hz, $^3J_{HH}$ = 6.5 Hz, 2H, 5,5'-position), 6.23 (d, $^3J_{HH}$ = 6.1 Hz, 2H, 5-cym-position), 5.99 (d, $^3J_{HH}$ = 6.1 Hz, 2H, 4-cym-position), 2.58 (sept, $^3J_{HH}$ = 6.9 Hz, 1H, 2-cym-position), 2.19 (s, 3H, 7-cym-position), 0.95 (d, $^3J_{HH}$ = 6.9 Hz, 6H, 1-cym-position).

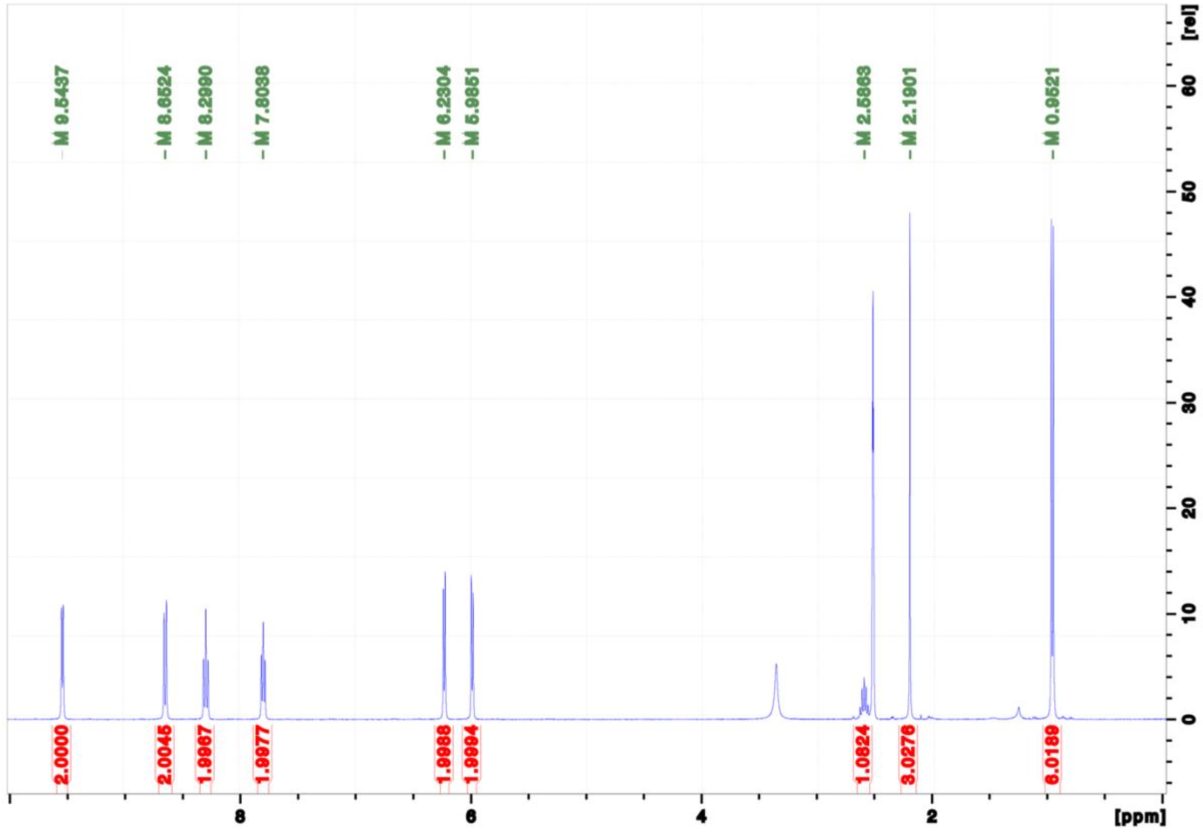

$^{13}C\{^1H\}$ NMR (100.57 MHz, d^6-DMSO): 156.2 (2,2'-position), 154.8 (6,6'-position), 140.4 (4,4'-position) 128.0 (5,5'-position), 124.3 (3,3'-position), 104.1 (3-cym-position), 104.0 (6-cym-position) 87.0 (5-cym-position), 84.3 (4-cym-position), 30.8 (2-cym-position), 22.1 (1-cym-position), 18.8 (7-cym-position).

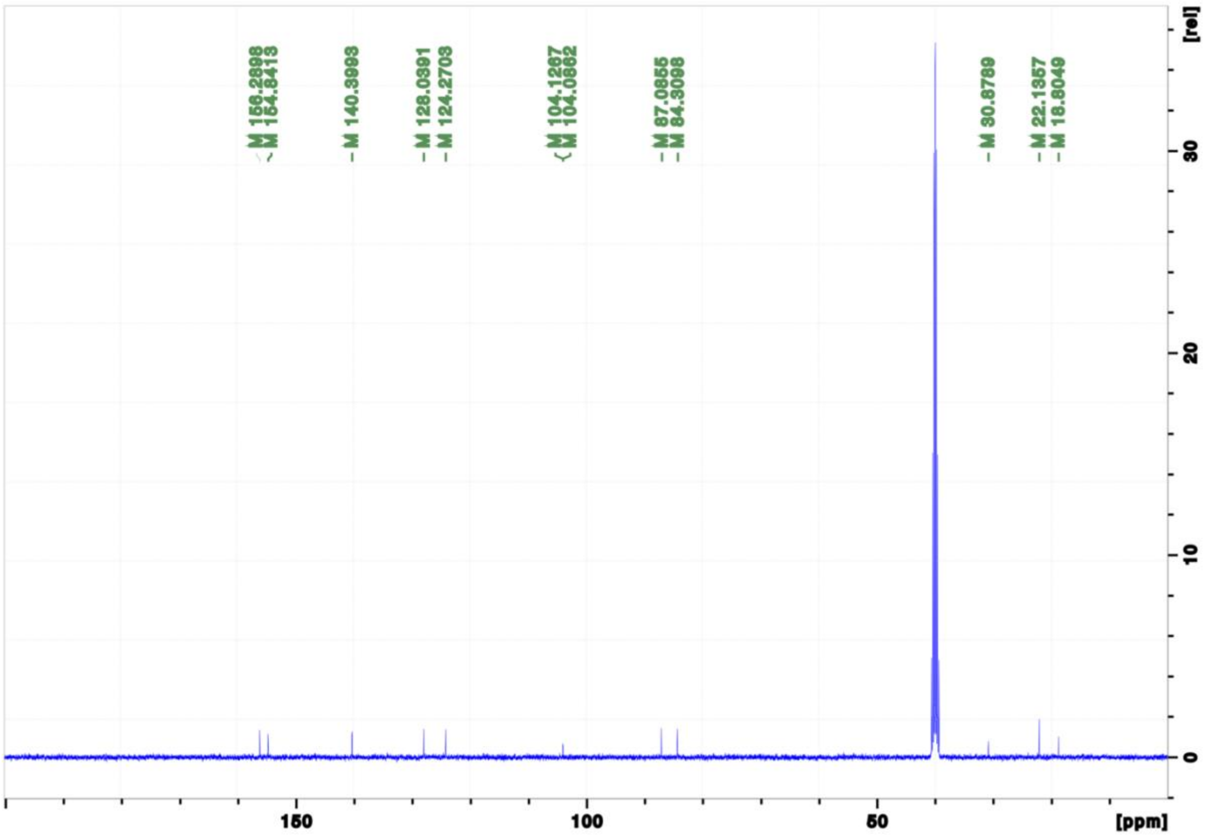

Characterisation of [Ru(η^6-*p*-cymene)(5,5'-difluorobipyridine)Cl][PF_6] – [2]

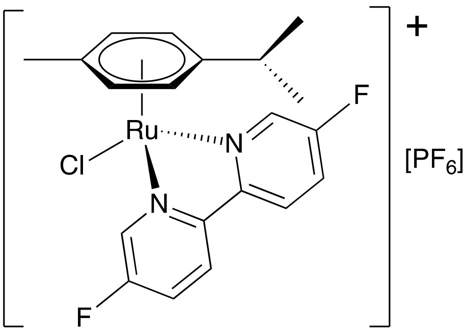

Yield: 59%. Recrystallised via vapour diffusion Et_2O:Acetone. Appearance: Orange needles

Elemental: Anal. Calcd for $C_{20}H_{20}ClF_8N_2PRu$: C, 39.52; H, 3.32; N, 4.61. Found: C, 39.73; H, 2.99; N, 4.12. **HRMS (ESI^+):** *m/z* 463.0370 [M – PF_6]$^+$ (m_{calc} = 463.0327). **^1H NMR** (400.13 MHz, d^6-DMSO): δ (ppm) 9.63 (overlapping dd, $^3J_{HF}$ = 3.2 Hz, $^4J_{HH}$ = 2.5 Hz, 2H, 6,6'-bipy-position), 8.71 (dd, $^3J_{HH}$ = 9.0 Hz, $^4J_{HF}$ = 4.8 Hz, 2H, 3,3'-bipy-position), 8.35 (td, $^3J_{HH}$ = 9.0

Hz, $^3J_{HF}$ = 8.0 Hz, $^4J_{HH}$ = 2.5 Hz, 2H, 4,4’-bipy-position), 6.33 (d, $^3J_{HH}$ = 6.4 Hz, 2H, 5-cym-position), 6.04(d, $^3J_{HH}$ = 6.4 Hz, 2H, 4-cym-position), 2.59 (sept, $^3J_{HH}$ = 7.0 Hz, 1H, 2-cym-position), 2.19 (s, 3H, 7-cym-position), 0.93 (d, $^3J_{HH}$ = 7.0 Hz, 6H, 1-cym-position).

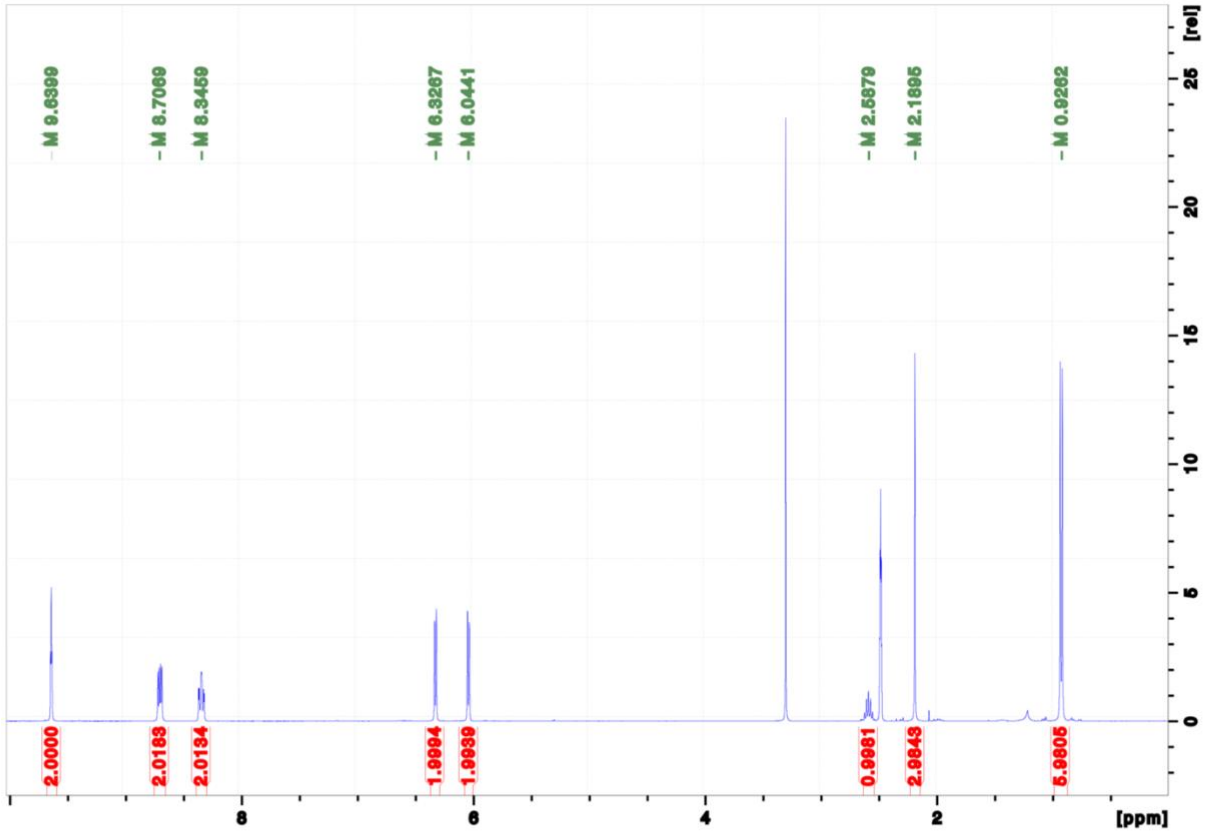

$^{13}C\{^1H\}$ NMR (100.57 MHz, d^6-DMSO): δ (ppm) 159.1 (d, $^1J_{CF}$ = 256 Hz, 5,5’-bipy-position), 150.5 (s, 2,2’-bipy-position), 144.5 (d, $^2J_{CF}$ = 32.5 Hz, 6,6’-bipy-position), 127.4 (d, $^2J_{CF}$ = 18.3 Hz, 4,4’-bipy-position), 125.3 (d, $^3J_{CF}$ = 8.0 Hz, 3,3’-bipy-position,), 105.0 (s, 3-cym-position), 104.4 (s, 6-cym-position), 86.8 (s, 5-cym-position), 83.5 (s, 4-cym-position), 30.4 (s, 2-cym-position), 21.7 (s, 1-cym-position), 18.4 (s, 7-cym-position).

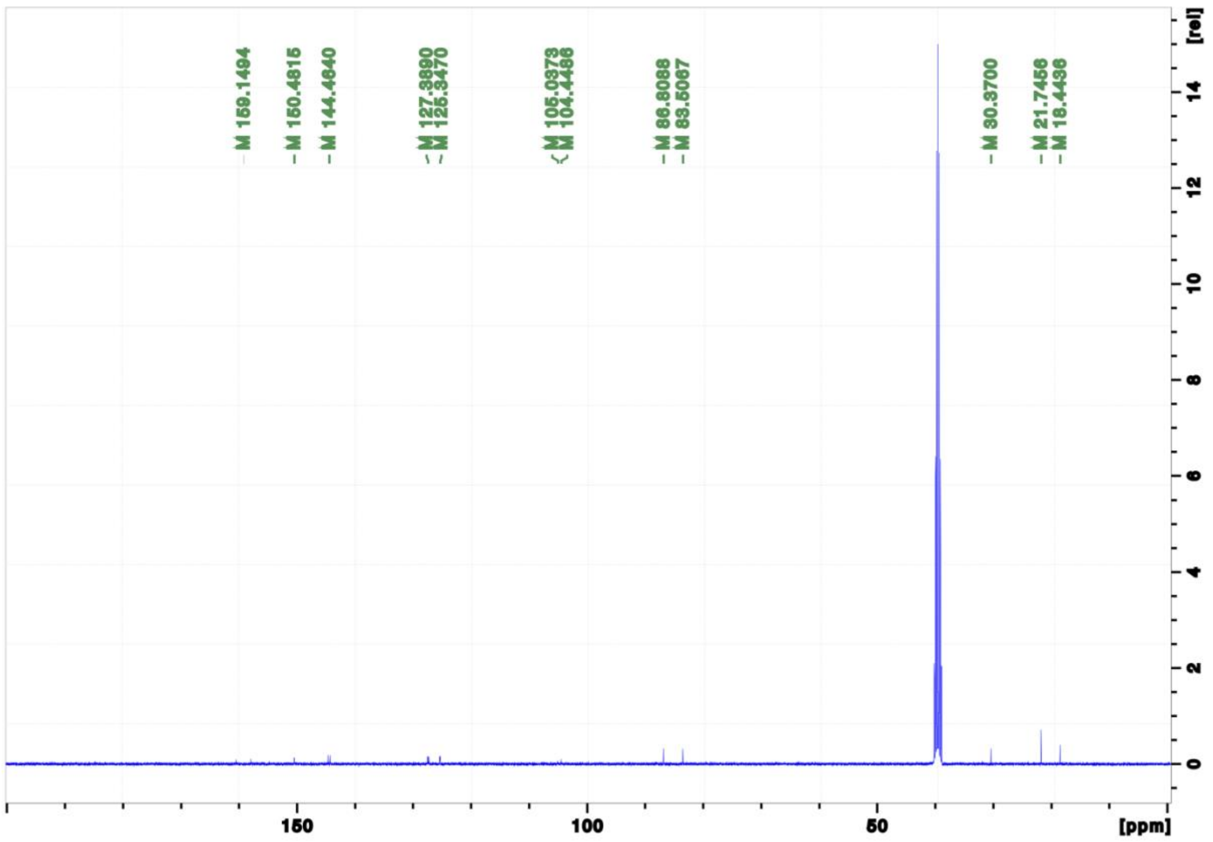

^{19}**F{^{1}H} NMR** (376.50 MHz, d^6-DMSO): δ (ppm) -70.1 d, $^{1}J_{PF}$ = 711 Hz PF_6), -119.2 (s, 5,5'-position).

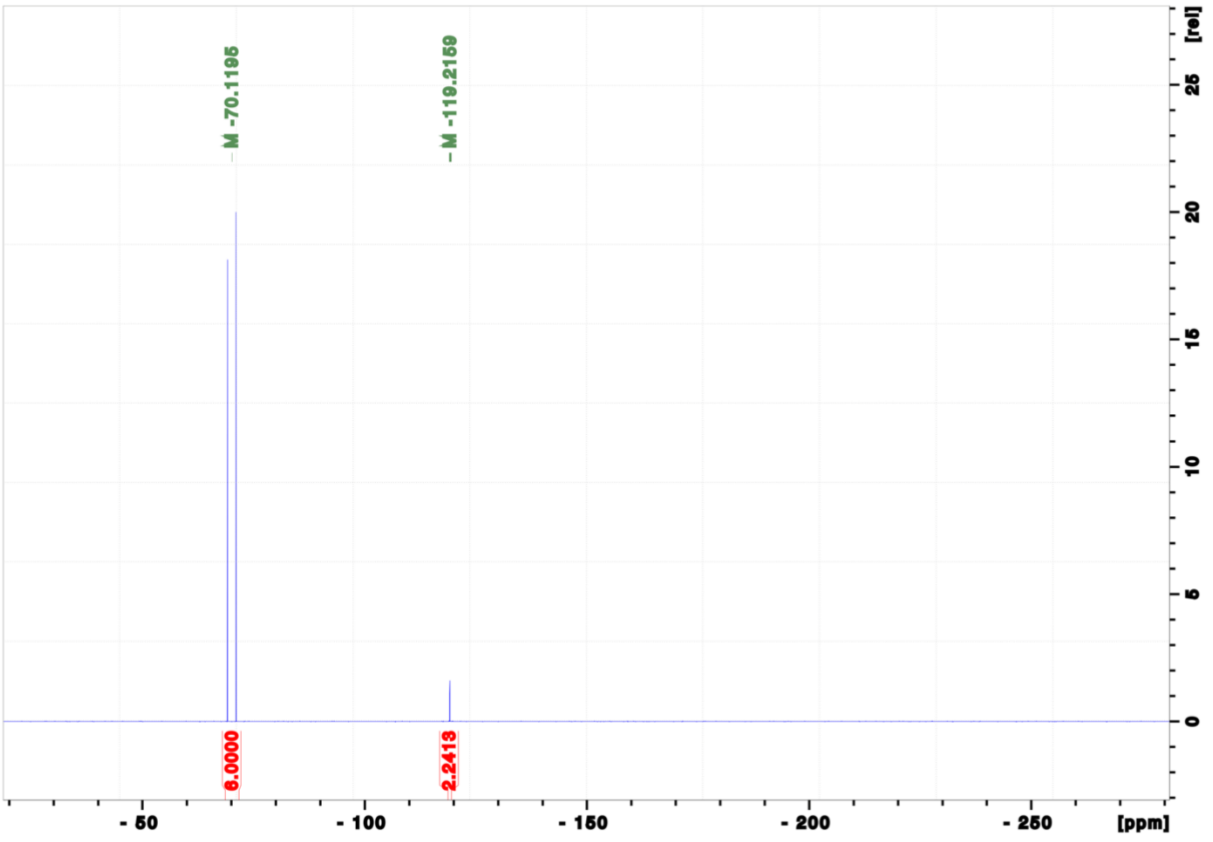

Characterisation of [Ru(η^6-*p*-cymene)(5,5'-di(trifluoromethyl)bipyridine)Cl][PF$_6$] – [3]

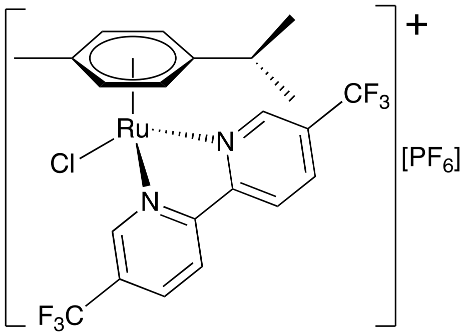

[15]

Yield: 62%. Recrystallised via vapour diffusion Et_2O:Acetone. Appearance: Yellow prisms.

Elemental: Anal. Calcd for $C_{22}H_{20}ClF_{12}N_2PRu$: C, 37.33; H, 2.12; N, 4.21. Found: C, 37.59; H, 2.71; N, 3.59. **HRMS (ESI$^+$):** *m/z* 563.0639 [M – PF_6]$^+$ (m_{calc} = 563.0263). ^{1}H NMR (400.13 MHz, d^6-DMSO): δ (ppm) 9.83 (s, 2H, 6,6'-position), 9.04 (d, $^3J_{HH}$ = 8.5 Hz, 2H, 3,3'-position), 8.86 (d, $^3J_{HH}$ = 8.5 Hz, 2H 4,4'-position), 6.45 (d, $^3J_{HH}$ = 6.1 Hz, 2H, 5-cym-position), 6.18 (d, $^3J_{HH}$ = 6.1 Hz, 2H, 4-cym-position), 2.62 (sept, $^3J_{HH}$ = 6.9 Hz, 1H, 2-cym-position), 2.21 (s, 3H, 7-cym-position), 0.97 (d, $^3J_{HH}$ = 6.9 Hz, 6H, 1-cym-position).

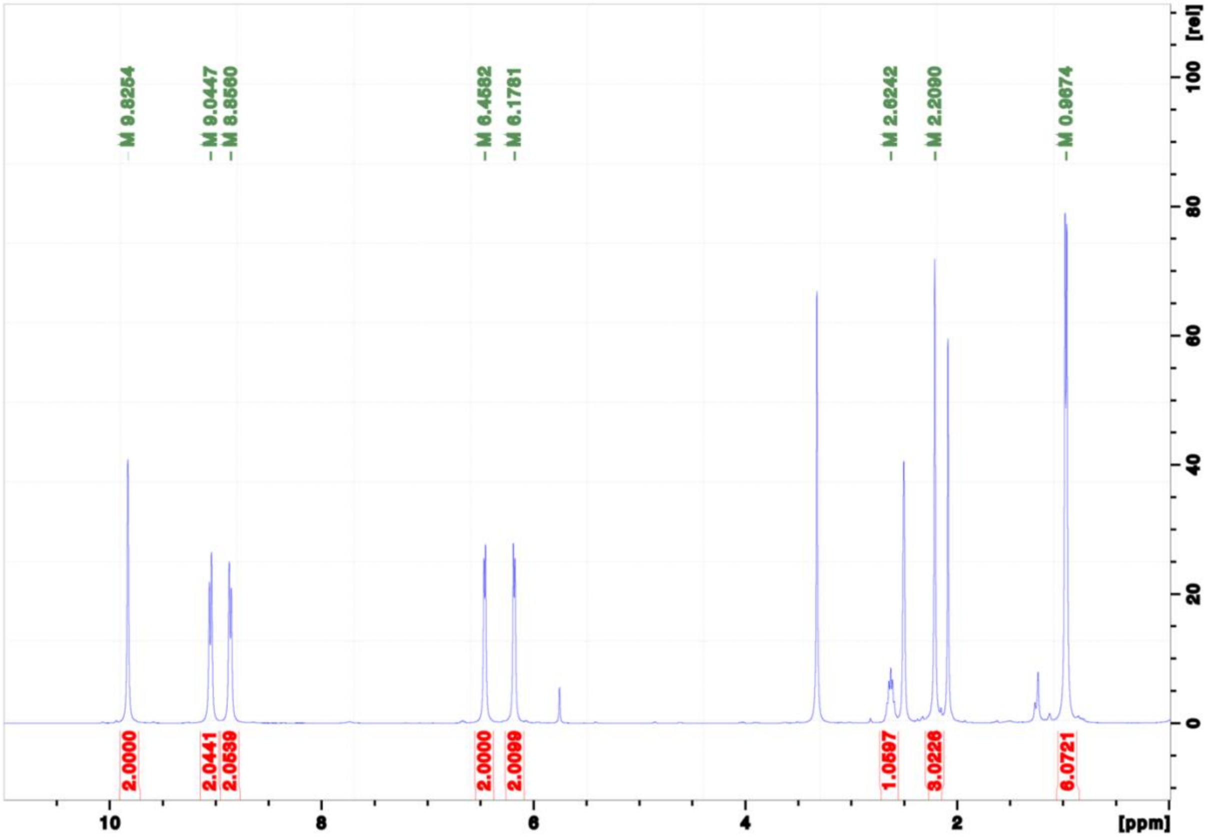

$^{13}C\{^{1}H\}$ NMR (100.57 MHz, d^6-DMSO): 156.5 (s, 2,2'-position), 152.3 (q, $^{3}J_{CF}$ = 4.2 Hz, 6,6'-position,), 137.8 (q, $^{3}J_{CF}$ = 3.3 Hz , 4,4'-position,), 128.9 (q, $^{2}J_{CF}$ = 34 Hz, 5,5'-position,), 125.7 (s, 3,3'-position) 122.0 (q, $^{1}J_{CF}$ = 273 Hz , <u>C</u>F_3,), 105.8 (s, 3-cym-position), 104.9 (s, 6-cym-position), 87.3 (s, 5-cym-position), 83.4 (s, 4-cym-position), 30.4 (s, 2-cym-position), 21.8 (s, 1-cym-position), 18.4 (s, 7-cym-position).

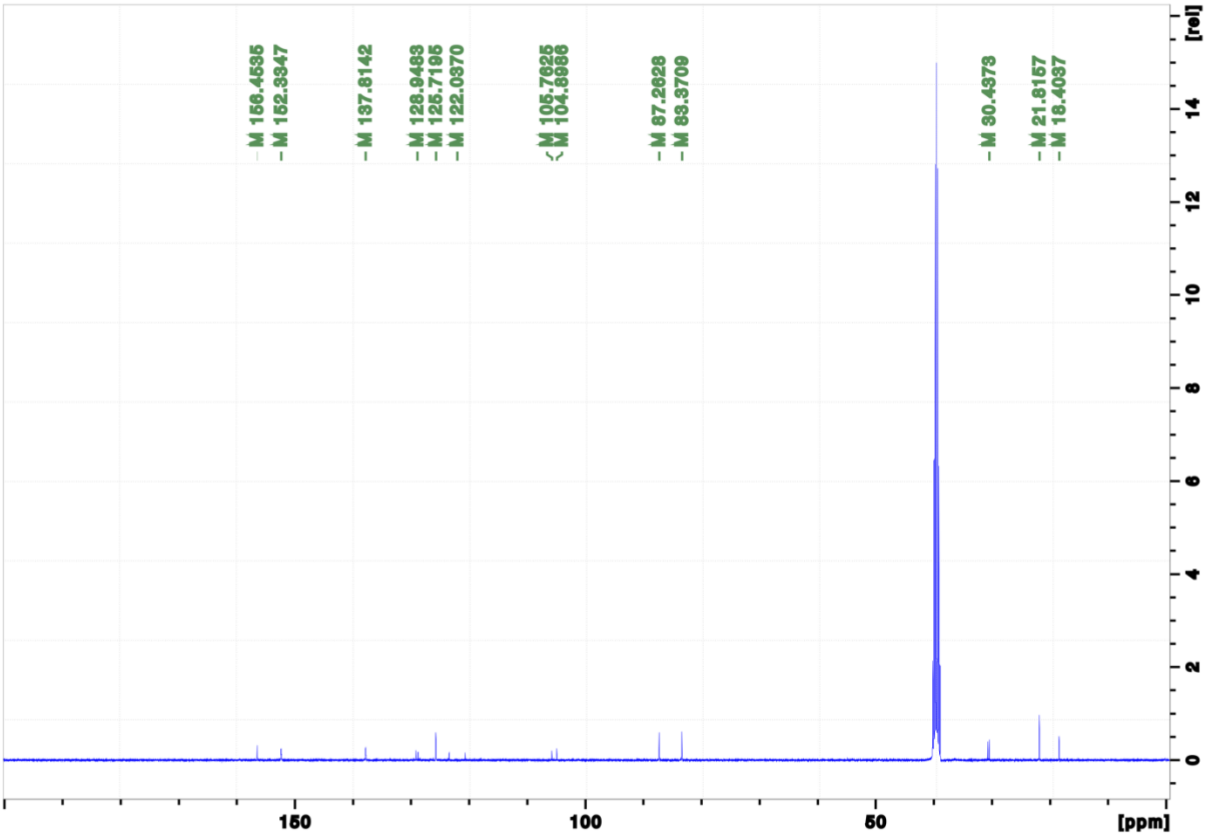

$^{19}F\{^{1}H\}$ NMR (376.50 MHz, d^6-DMSO): δ (ppm) -70.2 (d, $^{1}J_{PF}$ = 711 Hz, PF_6,), -60.4 (s, CF_3 groups).

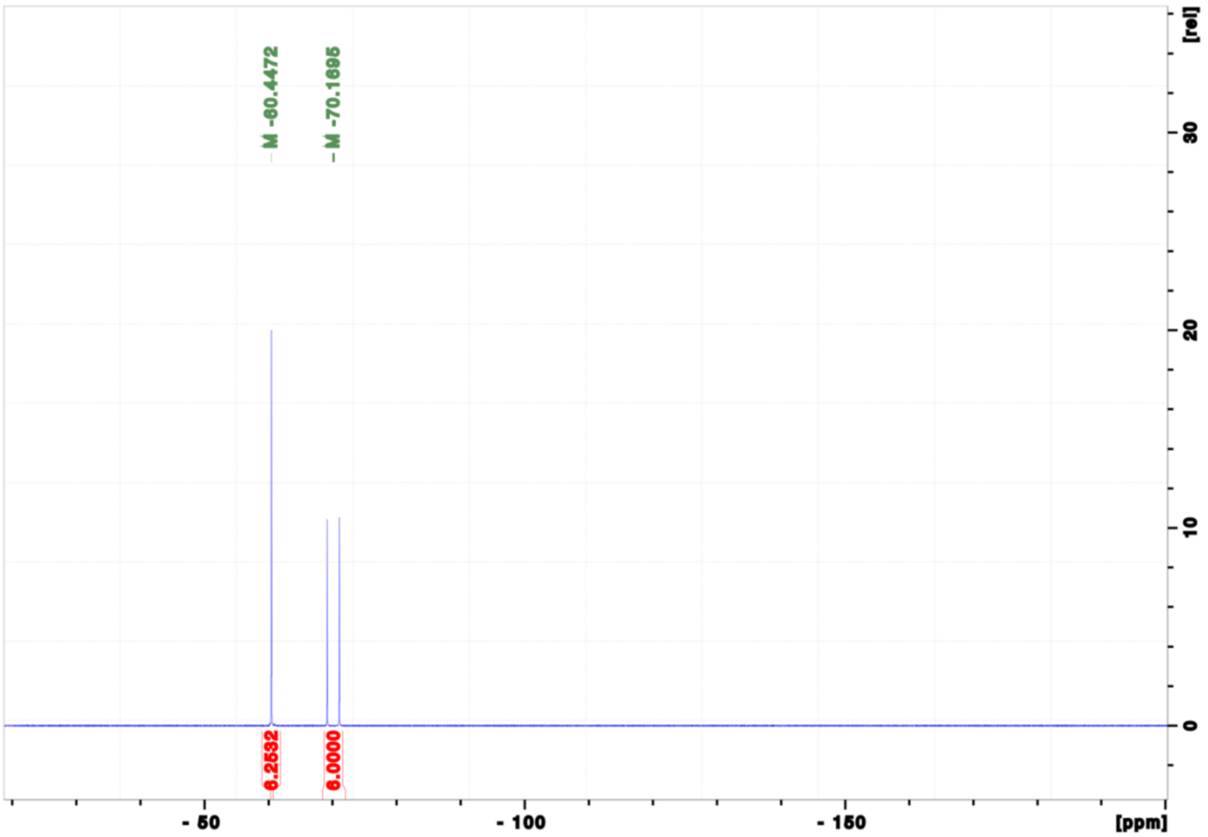

Characterisation of [Ru(η^6-hexamethylbenzene)(bipyridine)Cl][PF$_6$] – [4]

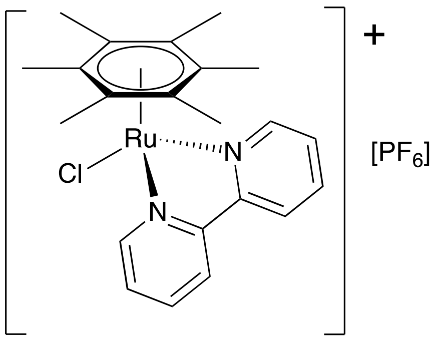

Yield: 45%. Recrystallised via vapour diffusion Et_2O:Acetone. Appearance: Orange prisms.

Elemental: Anal. Calcd for $C_{22}H_{26}ClF_6N_2PRu$: C, 44.04; H, 4.37; N, 4.67. Found: C, 44.18; H, 4.03; N 4.17. **HRMS (ESI$^+$):** *m/z* 454.0855 [M – PF_6]$^+$ (m_{calc} = 454.0828). **^{1}H NMR** (400.13 MHz, d^6-DMSO): δ (ppm) 8.92 (d, $^3J_{HH}$ = 5.7 Hz, 2H, 6,6'-position), 8.60 (d, $^3J_{HH}$ = 8.0 Hz, 2H, 3,3'-position), 8.24 (overlapping dd, $^3J_{HH}$ = 8.6 Hz, $^3J_{HH}$ = 8.0 Hz, 2H, 4,4'-position), 7.79 (overlapping dd, $^3J_{HH}$ = 8.6 Hz, $^3J_{HH}$ = 6.7 Hz, 2H, 5,5'-position), 2.02 (s, 18H).

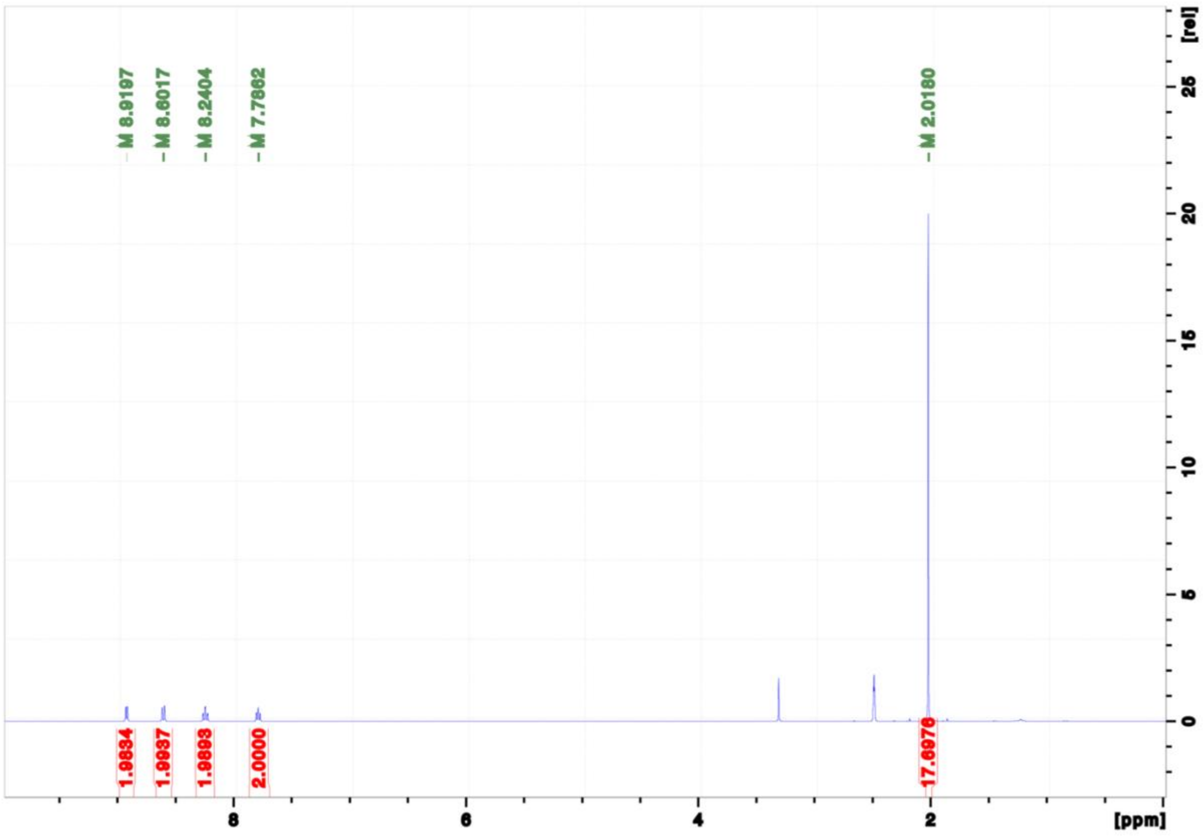

^{13}C{^{1}H} NMR (100.57 MHz, d^{6}-DMSO): 154.3 (2,2'-position), 153.7 (6,6'-position), 139.6 (4,4'-position) 127.7 (5,5'-position), 123.5 (3,3'-position), 95.5 (s, 2-hmb-position), 15.1 (s, 1-hmb-position).

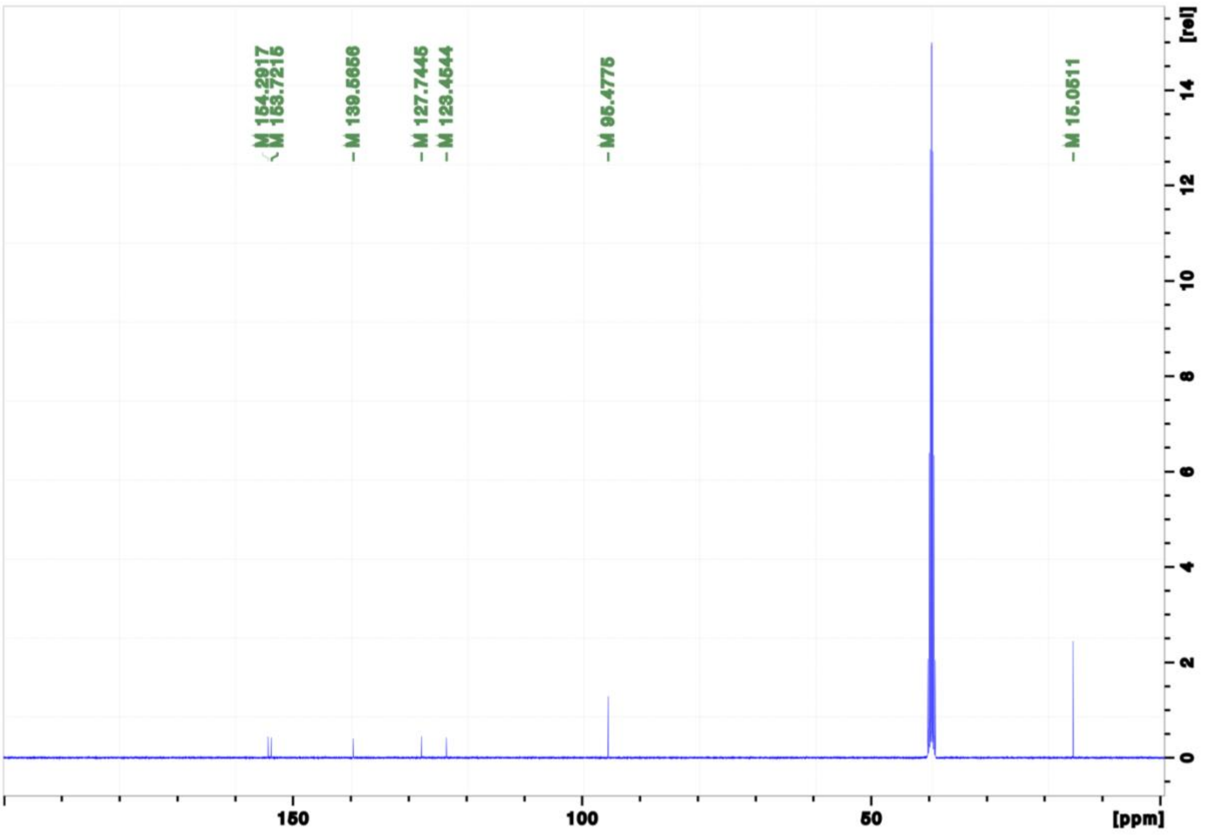

Characterisation of [Ru(η^6-hexamethylbenzene)(5,5'-difluorobipyridine)Cl][PF$_6$] – [5]

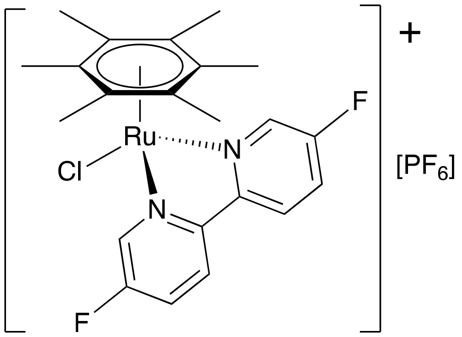

Yield: 59%. Recrystallised via vapour diffusion Et_2O:Acetone. Appearance: Red needles

Elemental: Anal. Calcd for $C_{22}H_{24}ClF_8N_2PRu$: C, 41.55; H, 3.80; N, 4.41. Found: C, 41.63; H, 3.46; N, 3.86. **HRMS (ESI$^+$):** *m/z* 491.0676 [M – PF_6]$^+$ (m_{calc} = 491.0640). **^{1}H NMR** (400.13 MHz, d^6-DMSO): δ (ppm) 8.89 (6,6'-bipy-position, 2H, t, $^3J_{HF}$ = 3.2 Hz, $^4J_{HH}$ = 2.3 Hz), 8.72 (3,3'-bipy-position, 2H, overlapping dd, $^3J_{HH}$ = 9.0 Hz, $^4J_{HF}$ = 4.8 Hz), 8.34 (4,4'-bipy-position, 2H, td, $^3J_{HH}$ = 9.0 Hz, $^3J_{HF}$ = 8.0 Hz, $^4J_{HH}$ = 2.3 Hz), 2.06 (18H, s).

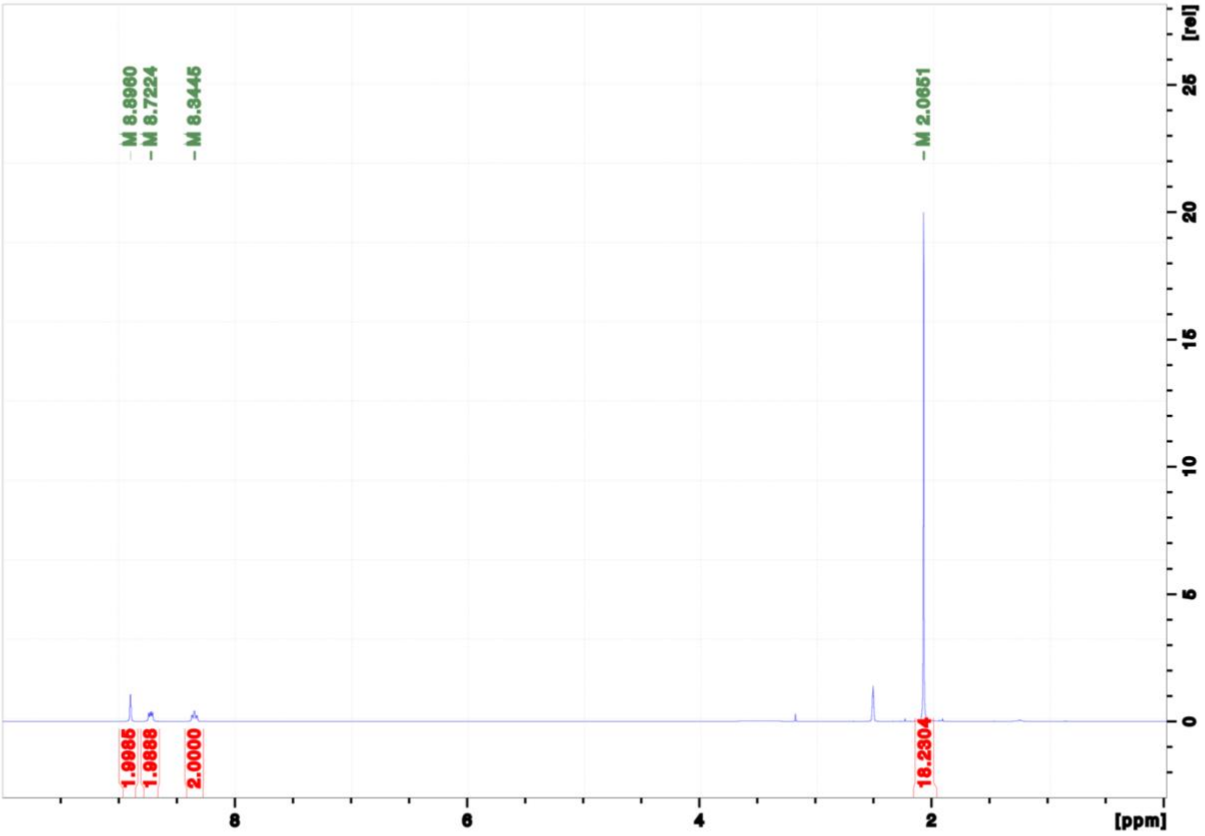

$^{13}C\{^{1}H\}$ NMR (100.57 MHz, d^6-DMSO): δ (ppm) 159.4 (d, $^{1}J_{CF}$ = 257 Hz, 5,5'-position), 150.6 (s, 2,2'-position), 141.7 (d, $^{2}J_{CF}$ = 32 Hz, 6,6'-position), 127.2 (d, $^{2}J_{CF}$ = 19.5 Hz, 4,4'-position), 125.2 (d, $^{3}J_{CF}$ = 8 Hz, 3,3'-position,), 95.8 (s, 2-hmb-position) 15.0 (s, 1-hmb-position).

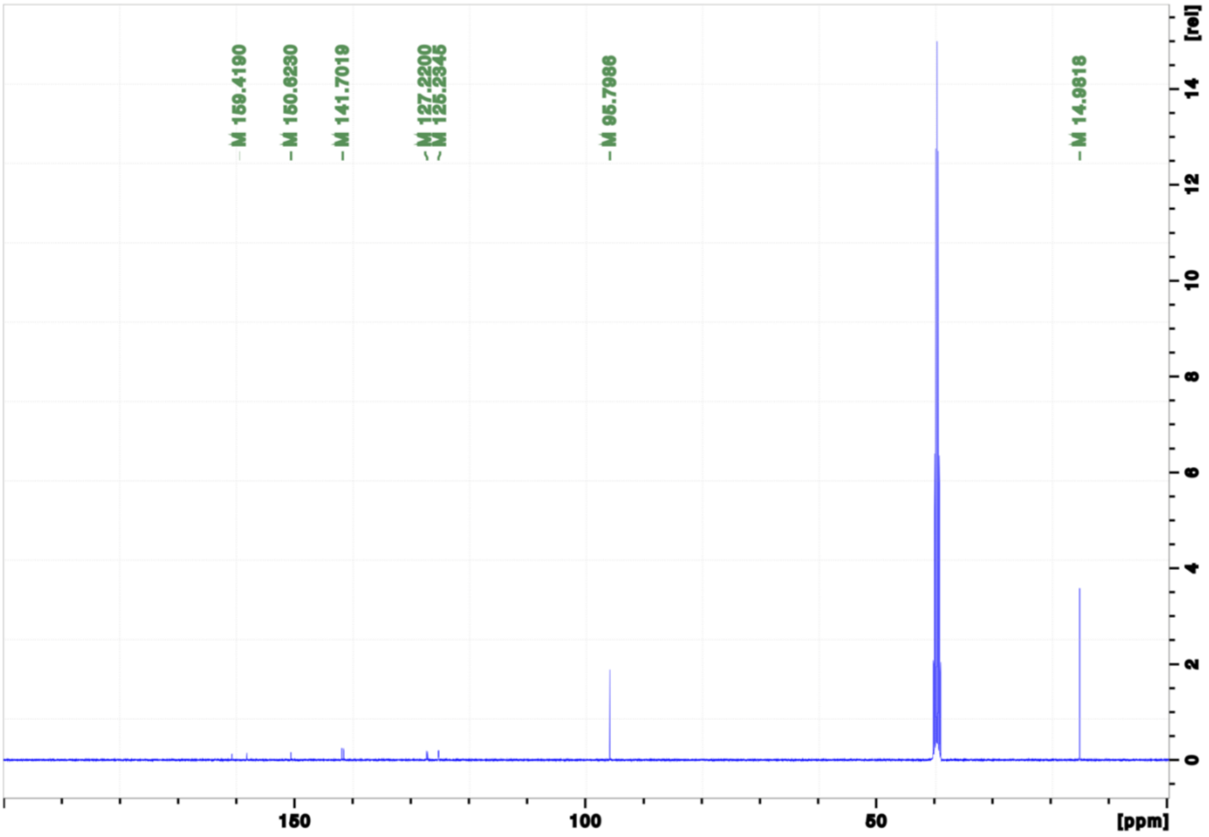

^{19}F{^{1}H} NMR (376.50 MHz, d^{6}-DMSO): δ (ppm) -70.2 (d, $^{1}J_{PF}$ = 711 Hz, PF_6), -118.9 (s, 5,5'-position).

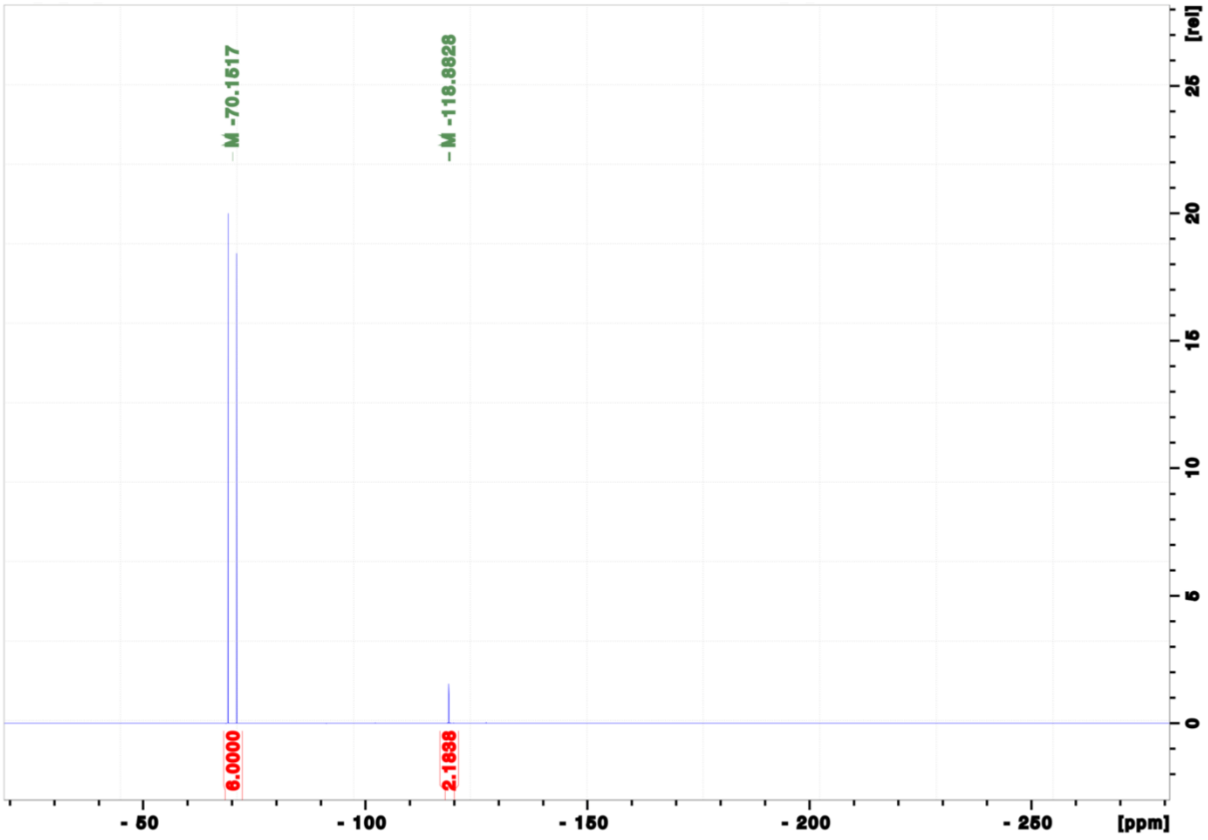

Characterisation of [Ru(η^6-hexamethylbenzene)(5,5'-di(trifluoromethyl)bipyridine)Cl][PF_6] – [6]

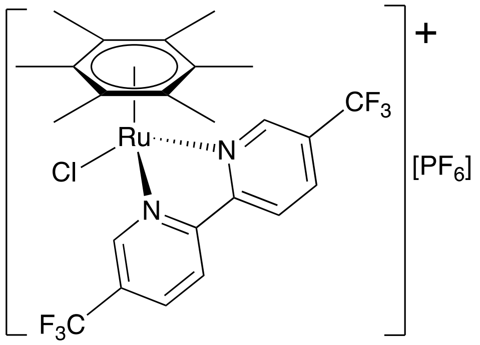

Yield: 45%. Recrystallised via vapour diffusion Et_2O:Acetone. Appearance: Orange Needles.

Elemental: Anal. Calcd for $C_{22}H_{20}ClF_{12}N_2PRu$: C, 39.17; H, 3.29; N, 3.81. Found: C, 38.94; H, 2.93; N, 3.50. **HRMS (ESI$^+$):** *m/z* 591.0573 [M – PF_6]$^+$ (m_{calc} = 591.0576). **^{1}H NMR (**400.13 MHz, d^6-DMSO): δ (ppm) 9.16 (s, 2H, 6,6'-position), 9.08 (d, $^3J_{HH}$ = 8.4 Hz, 2H, 3,3'-position), 8.88 (d, $^3J_{HH}$ = 8.4 Hz, 2H 4,4'-position), 2.08 (18H, s).

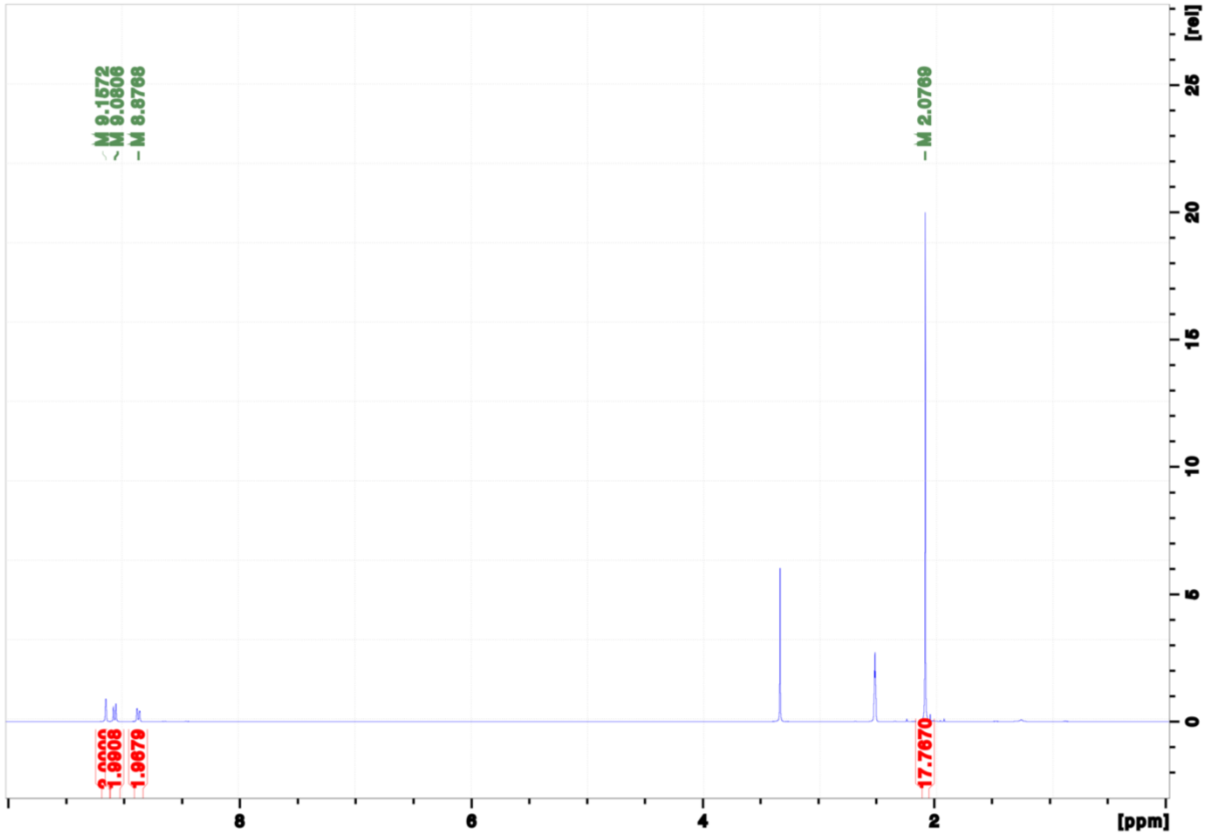

^{13}C{^{1}H} NMR (100.57 MHz, d^6-DMSO): 156.6 (s, 2,2'-position), 149.4 (q, $^3J_{CF}$ = 4.3 Hz, 6,6'-position), 137.5 (q, $^3J_{CF}$ = 2.9 Hz , 4,4'-position,), 128.7 (q, $^2J_{CF}$ = 34 Hz, 5,5'-position,), 125.7

(s, 3,3'-position) 122.2 (q, $^{1}J_{CF}$ = 274 Hz , $\underline{C}F_{3}$,), 96.2 (s, 2-hmb-position) 15.0 (s, 1-hmb-position).

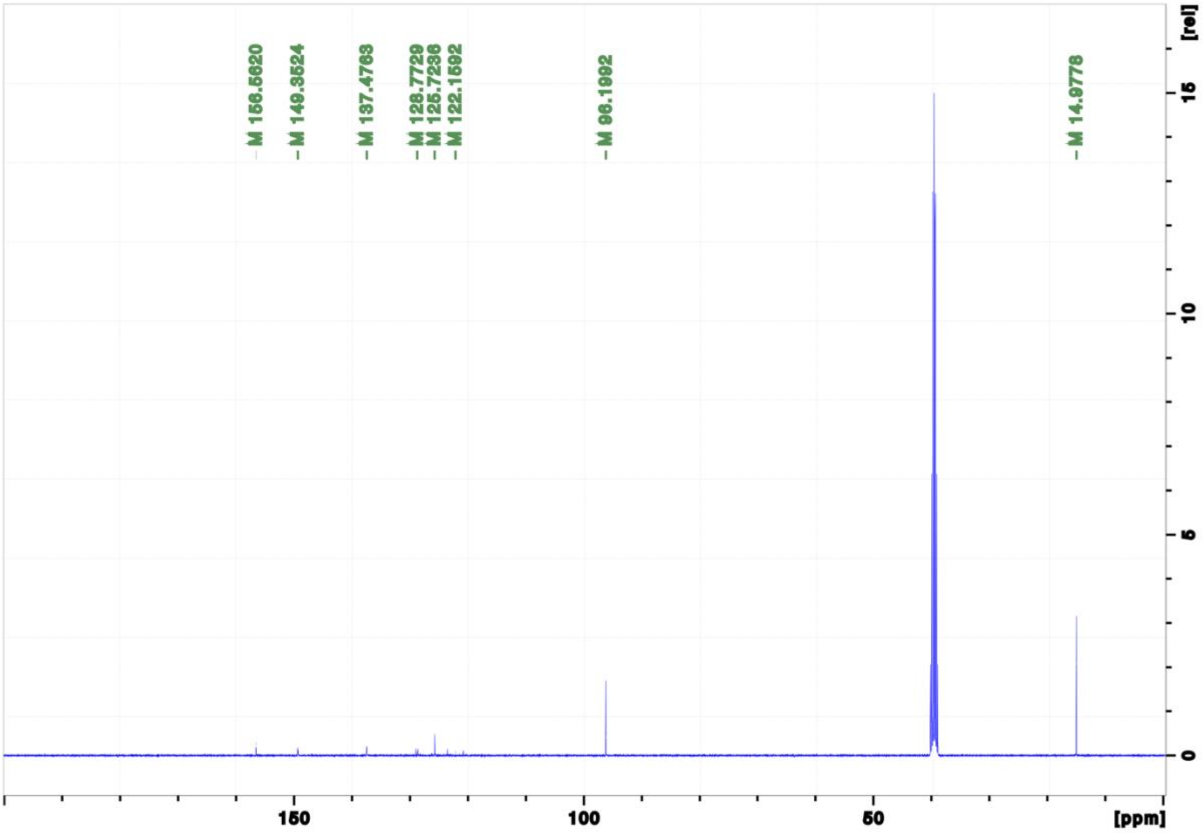

$^{19}F\{^{1}H\}$ NMR (376.50 MHz, d^{6}-DMSO): δ (ppm) 70.2 (d, $^{1}J_{PF}$ = 711 Hz, PF_{6},), -60.8 (s, CF_{3} groups).

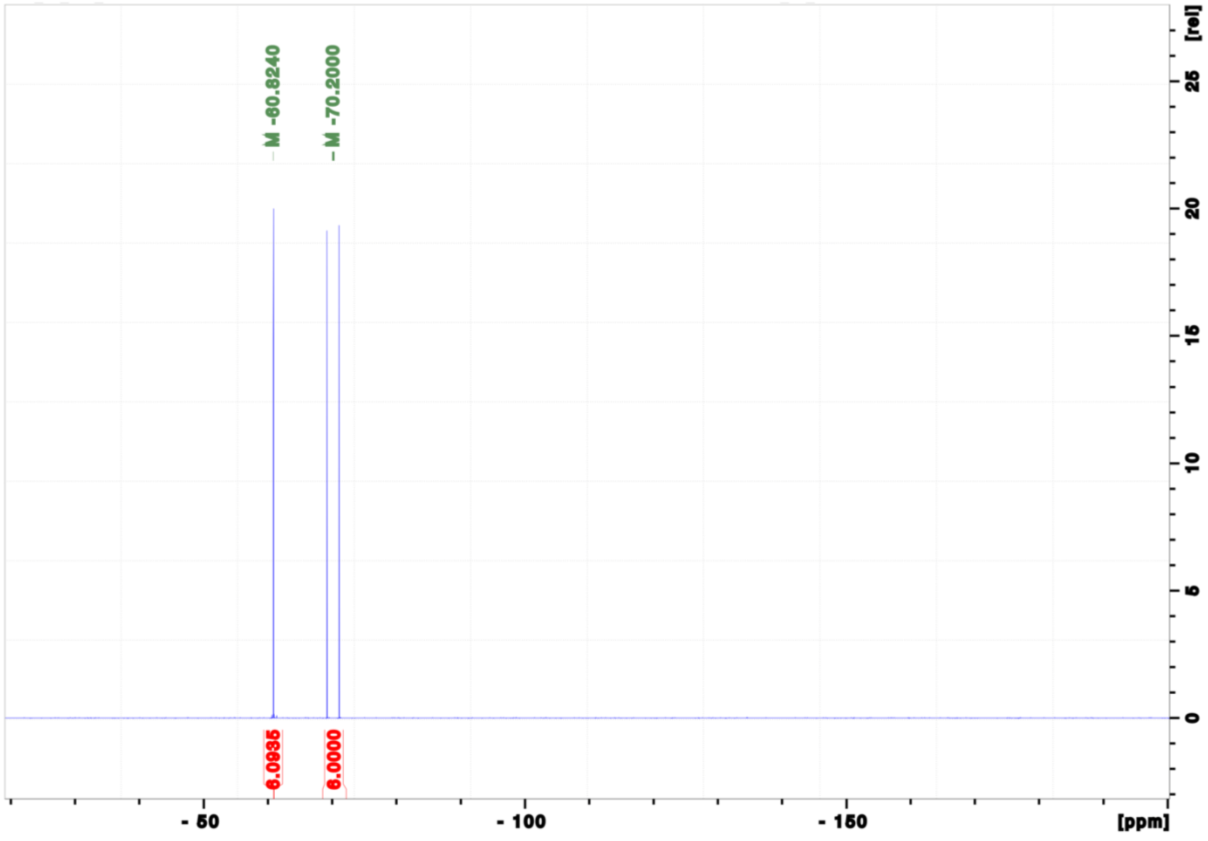
M -60.8240
M -70.2000
6.0935
6.0000
[rel]
25
20
15
10
5
0
- 50
- 100
- 150
[ppm]

References

(1) Barker, P. D.; Nerou, E. P.; Freund, S. M. V.; Feamley, I. M. *Biochemistry* **1995**, *34* (46), 15191.

(2) Garcia, P.; Bruix, M.; Rico, M.; Ciofi-Baffoni, S.; Banci, L.; Shastry, M. C. R.; Roder, H.; de Lumley Woodyear, T.; Johnson, C. M.; Fersht, A. R.; Barker, P. D. *J. Mol. Biol.* **2005**, *346* (1), 331.

(3) Keller, A.; Nesvizhskii, A. I.; Kolker, E.; Aebersold, R. *Anal. Chem.* **2002**, *74* (20), 5383.

(4) Lee, K.; Lee, P. H. *Tetrahedron Lett.* **2008**, *49* (27), 4302.

(5) Zhao, J.; Rebelein, J. G.; Mallin, H.; Trindler, C.; Pellizzoni, M. M.; Ward, T. R. *J. Am. Chem. Soc.* **2018**, *140* (41), 13171.
